# Spatiotemporal modelling and monitoring of harmful algal blooms using IoT in Lake Victoria Basin Kenya

**DOI:** 10.1038/s41598-025-21979-3

**Published:** 2025-10-30

**Authors:** Jacob Okello Okomo, Eunice Nduati, Fridah Kirimi

**Affiliations:** Department of Geomatic Engineering and Geospatial Information System, Jomo Kenyatta University of Agriculture and Technology, Nairobi, Kenya

**Keywords:** Harmful algal blooms (HABs), Internet of things (IoT), Automated in situ sensors, Environmental impact, Hydrology

## Abstract

The escalating global concern of harmful algal blooms (HABs) in aquatic environments, poses significant challenges in the context of sustainable development goals (SDGs). The impacts of HABs extend beyond increased waterborne diseases, causing high aquaculture mortality, diminished aesthetic appeal, elevated drinking water treatment costs, and negative effects on tourism and gross domestic product (GDP). Traditional HAB assessment methods involving field sampling and laboratory analysis are inefficient due to their labor intensiveness and cost. This study introduces a novel approach, combining near real-time satellite remote sensing with a low-cost *in-situ* internet of things (IoT) system that adds a layer of real-time data collection and timely reporting. The method utilizes the ocean color algorithm and the mono-window lake surface air temperature (LSAT) algorithm, enabling widespread detection and mapping of cyanobacteria-induced HABs with a 30 m spatial resolution landsat imagery. The research involved deployment of immobile *in-situ* IoT system for continuous near real-time LSAT monitoring at select locations which were as well Kenya marine and fisheries research institute (KMFRI) HAB sampling sites that were prone to early HAB occurrence. From 2015 to 2020, the study used Landsat 8 data to monitor chlorophyll-a (Chl-a) concentrations as proxies for HABs. The Ocean Colour 2 algorithm, combined with landsat 8 thermal infrared (TIR) sensor data, estimated LSAT during blooms. Results are validated against datasets from Copernicus’ Sentinel-3 and national aeronautics and space administration’s (NASA) moderate resolution imaging spectroradiometer (MODIS) missions. Findings showed significant increases in Chl-a values (31 to 57.1 Mg/M^3^) and LSAT (35.1 to 36.6 °C) during blooms, while unaffected areas had lower Chl-a concentration values ranging from (−1.2 to 16.4 mg/m^3^) and corresponding lower LSAT values of (16.9 to 28.7 °C). Validation using sentinel-3 ocean and land colour instrument (OLCI) and MODIS data confirmed the algorithm’s reliability with coefficients of determination (R^2^) ranging from 0.837 to 0.899 for sentinel 3-OLCI and 0.667 to 0.821 for MODIS datasets. An automated IoT powered in-situ system for LSAT monitoring reported abnormally higher average temperature rises (to the above scores, against the normal 25.4 °C), for potential real-time HAB alerts.

**Supplementary Information:**

The online version contains supplementary material available at 10.1038/s41598-025-21979-3.

## Introduction

Algal blooms, characterized by the rapid growth of one or more species leading to an increase in biomass, have gained global attention, especially harmful algal blooms (HABs) associated with toxic species like *Cyanotoxins spp*^1,2^.. These phenomena, often referred to as “Red Tides,” pose environmental and health risks^3^, emphasizing the need for effective detection and monitoring methods, thus have attracted significant world-wide attention in research over the last two decades^4,5^.

In retrospect, before the advent of active satellite remote sensing, locals inhabiting the littoral zones of Lake Victoria have reportedly relied on traditional means to assess the HABs including visual observations of unusual water color & turbidity arising from either greenish or brownish tint created by the dense mats of scum on the water surface which suddenly realizes unusual massive fish deaths^6^.

Multiple approaches have since been employed to monitor water quality and HABs, each with trade-offs in resolution, scalability and operational feasibility. Traditional methods such as spectrometry and high-performance liquid chromatography (HPLC) offer precise quantification of Chl-a, nutrients and *Cyanotoxins* but are resource-intensive and limited in spatial and temporal coverage^7^. *In-situ* buoy-based array of sensors facilitate continuous, real-time measurement of physicochemical parameters but are limited to point locations and entail high maintenance costs^8^. Hyperspectral imaging via aerial platforms offers high spatial-resolution mapping of HABs but are constrained by high operational costs and limited spatial extent^9^. This study therefore integrates satellite remote sensing with *in-situ* IoT networks to enable scalable, cost-efficient, and near real-time HAB surveillance across broad spatial domains.

Numerous geoscientists have proposed approaches for HAB detection, including spectral band ratio algorithms using various satellite sensors. While existing methods focus on sensors like MODIS and Sea-viewing Wide Field-of-view Sensor (SeaWiFS), the study underscores the potential of Landsat 8 OLI, with its finer spatial resolution (30 m), for Chl-a retrieval^4,10,11^. Additionally, Landsat 8’s Thermal Infrared (TIR) data, with its ability to penetrate the often cloudy Lake Victoria region, is crucial for assessing lake surface air temperature (LSAT) during blooms^12^. The integration of Internet of Things (IoT,which is a network of interconnected physical devices loaded with water quality monitoring sensors and other technologies that monitor, collect and autonomously exchange data over the network, facilitating near real-time monitoring of the proxies of HABs, such as Lake Surface Air Temperature (LSAT, unusual turbidity, pH levels, Lake Salinity, crucial for HAB detection^13,14^.

Harmful algal blooms present significant environmental, health, and economic challenges globally, with a recent surge in documented cases^15^. Environmental consequences include the depletion of dissolved oxygen, impacting aquatic life and fisheries^16^. Human and animal health risks arise from toxic reactions to seafood, while economic threats take a toll on coastal industries like fishing and tourism^17^.

This study therefore aims to leverage Landsat 8’s high spatiotemporal resolution and IoT systems for improved HAB detection and monitoring in Lake Victoria, keeping a close focus on key proxies like Chl-a concentration, Lake Surface Air Temperatures, among others like phycocyanin levels, turbidity, Secchi depth and pH level^18^. This study focuses on the first two parameters, while recommends for the rest.

The study zone, Nyanza Gulf of Lake Victoria, faces deteriorating water quality and signs of eutrophication^19^. Despite these threats, a crucial gap exists in timely notification to authorities, hindering prompt responses. Current reliance on citizen reports leads to delayed responses and exacerbates the impacts of HABs.

## Research objectives

The main objective of this research is to detect, monitor and report the occurrence of Harmful Algal Blooms (HABs) and Cyanobacteria in Lake Victoria. This is achievable through the following specific objectives:

1. Monitoring the concentration of chlorophyll-a (Chl-a) concentrations from L8 OLI data.
2. Monitoring Lake Surface Air Temperature (LSAT) from L8 TIRS images
3. To develop and deploy an automated Internet of Things (IoT) in situ system, applicable in near real-time to monitor and report geo-tagged Water quality data (e.g., LSAT).

The following questions are formulated with respect to the objectives:

1. Can space based observation systems be used to detect and monitor HABs in Lake Victoria Inland water body?
2. Does HAB occurrence have a direct impact on the LSAT and LSWT at their point of influence?
3. Can IoT be utilized to monitor HAB occurrences inland water Lakes in near real-time?

## Materials and methods

### Study area

Lake Victoria, with an extensive surface area of about 69,484 KM^20^ and an average depth of 40 m at a maximum depth of 79 m ranks the second largest freshwater lake in the world after Lake Superior and the largest in Africa. Lying between 3° S to 0° 30’ N latitude and 31° 40’ E to 34° 50’E longitude is distributed among these three East African countries viz Tanzania 51%, Uganda 43%, and Kenya the remaining 6%^20,21^.

That in place, the lake is privileged to serve as the economical home of over 42 million residents^22,23^ in those riparian reserves. These millions of individuals solely bank on the lake for all aspects of their daily economic livelihood ranging from fishing, agriculture, and industrial applications just to barely highlight but a few. In that regard, its ecological monitoring should be of great geoscientific interest.

Lake Victoria is in Equatorial regions of the globe, and thus has an alternating climatic condition varying from tropical rain forest with rainfall over the lake for a better portion of the year to a semi-dry climate with sporadically discontinuous droughts over some locations^24^. The climatic conditions provide ambient temperatures varying between 18–26 °C which provide optimum host conditions for the growth and development of the *Cyanobacteria spp.*^1^.

The peripheral lands engulfing the study area is characterized by diverse soil types, predominantly ferrosals and acrisols, weathered soils rich in iron and aluminum oxides that realize moderate fertility for agricultral activities which are inherently common in tropical environments^25,26^. The surrounding land use and land cover consist of mixed subsistence agriculture, urban settlements, wetlands and patches of natural vegetation. Notably, moderately heavy agricultural activities that dominate the uplands, papyrus-dominated wetlands and aquatic vegetation that fringe the lake edges, significantly influence nutrient loading and runoff dynamics relevant to HAB development^27,28^.

**Fig. 1:**
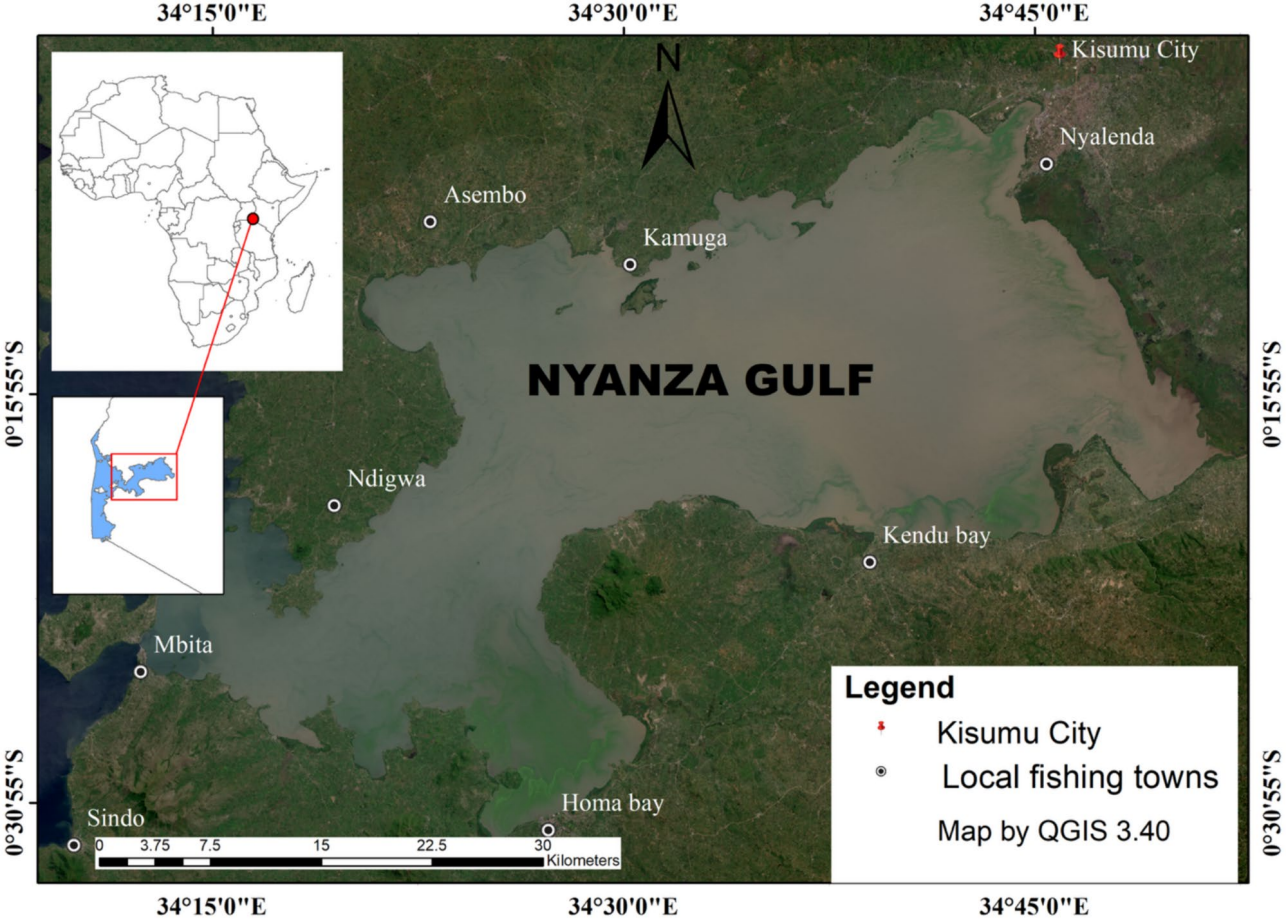
Map of Winam Gulf, Lake Victoria with study sites. Map created by authors using QGIS 3.40 (https://qgis.org).

Figure 1 shows the location and extent of the extract of the study area particularly relevant to this study.

### Data

#### Landsat 8 OLI/TIRS

NASA’s Landsat data from two instruments, namely the operational land imager (OLI), and thermal infra-red sensor (TIRS) were used. The landsat 8 operational land imager sensor was applied from where spectral bands 2 to 5 centered on Blue (480 nm), Green (560 nm), Red (655 nm) to generate true colour composites (TCC) and NIR (865 nm) was used in the extraction of Chl-a. Thermal data extracted from the L8 TIR sensor from which TIR1 (10,895 nm) band 10 was used for the extraction of LSAT.

Data obtained from the Kenya marine and fisheries research institute (KMFRI)^29^ department, NASA’s earth explorer^30^ and African Great Lakes (AGL)^20^ which entailed the reported HAB dates, as seen in Table 1 below, which were cross-referenced and validated with the Satellite remote sensing data for Chl-a on the reported dates, from 2015 to 2021.

**Table 1:** HABs reported in Lake Victoria, (KMFRI, RCMRD, NASA earth data).

Year	Date	Source	Sensor	Satellite	Product
2015	12^th^ January	NASA	OLI/TIRS	Landsat-8	TCC, Chl-a
	22^nd^ February	KMFRI	In-situ sampling, fishermen reports, Unusual massive fish kills		Chl-a, Turbidity
2016	23^rd^ February	KMFRI			Foul odor, greenish water color & scum
2017	04^th^ September	AGL	In-situ sampling, fishermen reports, Unusual massive fish kills, HAB Colonies		Chl-a, Turbidity
2018	27^th^ January	KMFRI			Foul odor, greenish water color
	27^th^ January	NASA	OLI/TIRS	Landsat-8	TCC, Chl-a
2019	18^th^ August	KMFRI	In-situ sampling, fishermen reports, Unusual massive fish kills, High surface temperatures		Photo evidence, Scum layers
2020	29^th^ August	KMFRI			Cl-a, pH
2021	No data	No data			

GIS shape-files were used for the convenient delineation of study areas.

For the purpose of ground-truthing & continuous observation & monitoring, in-situ data, in particular the Lake Surface Air Temperature from the locally deployable autonomous IoT sensors were collected.

### Methodology

Chl-a exhibits high absorption behavioral patterns around the blue (450–475 nm) and red (650-675 nm) wavelengths of L8. Further, at the green and NIR regions of the EM spectrum, Chl-a exhibits high reflectance values that could reach 500 and 700 nm, respectively^31^. The presence of narrower bandwidths and at a finer spatial resolution of 30 m improves L8’s pigment discrimination ability even in water bodies even though it was purposely designed for terrestrial applications^32,33^. Further, the second Thermal InfraRed (TIR)sensor on board L8 provides for the measuring of the Lake Surface Air Temperature^33^ which is another significant proxy for HABs that is investigated in this study.

#### Satellite data preprocessing

The Landsat 8 images used in this study were sourced from the U.S. Geological Survey (USGS) Earth Explorer^30^, specifically from Collection 2, which provides improved geometric accuracy and radiometric calibration^34^. These images are Level-2 surface reflectance products, processed to account for atmospheric effects and provide bottom-of-atmosphere reflectance values suitable for chlorophyll-a (Chl-a) retrieval.

**Fig. 2:**
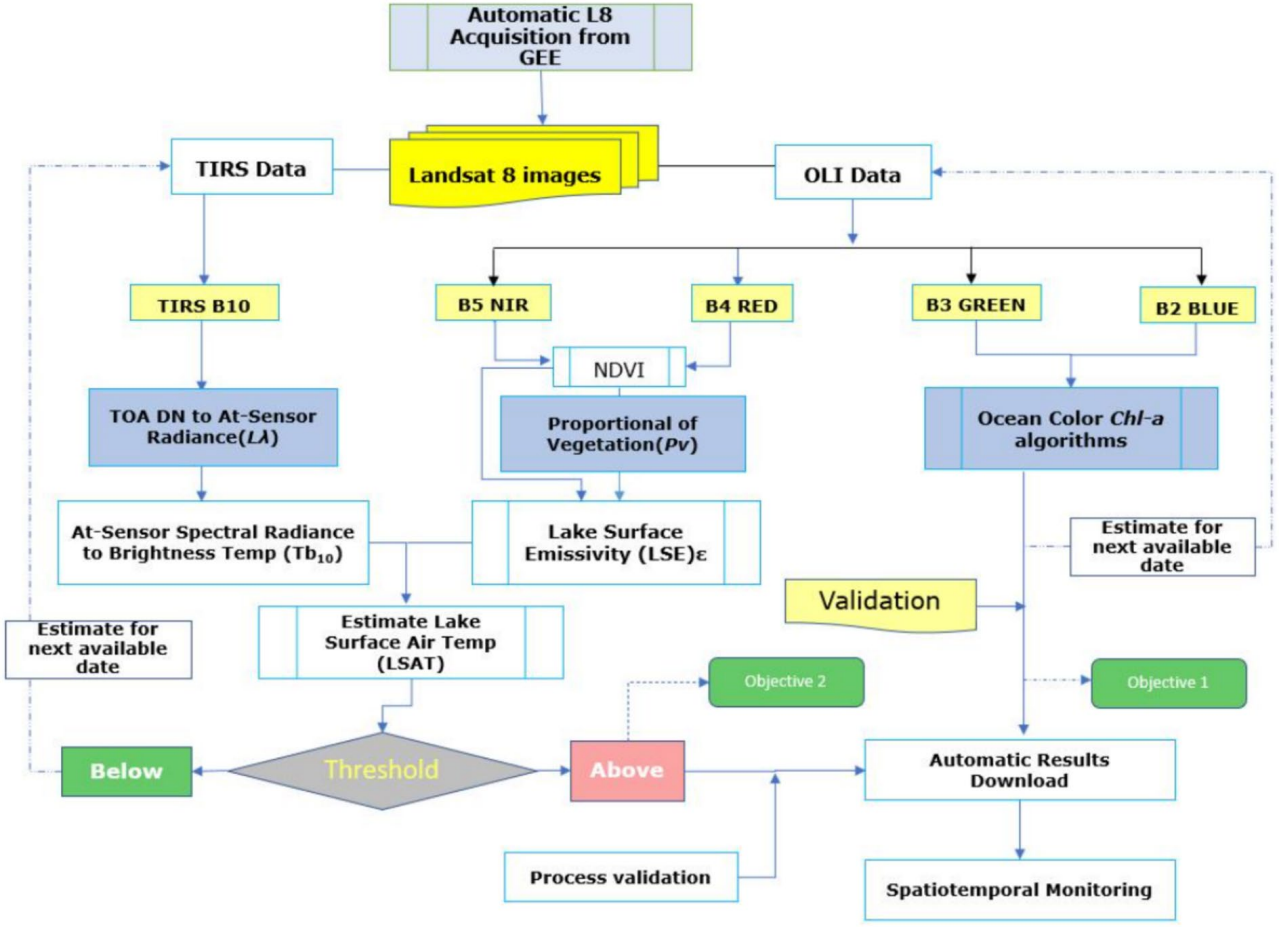
Overall methodology workflow for objectives 1 and 2.

Retrieval of Chl-a from Landsat-8 sensor over the study region involved the following three major steps figuratively illustrated in the methodological workflow in Fig. 2 below,

1. Obtaining absolute scaled DN values for all the required bands (B2, B3, B4 and B5).
2. Atmospheric correction of the images to minimize the atmospheric attenuation effects in the quite humid Lake Victoria regions.
3. Chl-a were retrieved by utilizing ocean chlorophyll (OC) algorithms (OC-2 shown in Eq. 1 below).

#### Estimating Chl-a concentrations

OC-2 is a modified fourth order polynomial algorithm which was originally developed for the SeaWiFS data^35,36^ but can be tuned to perform relatively well with Landsat 8^37,38^.

Mathematically, OC-2 is an empirical two-band ratio algorithm^34^ for the quantitative estimation of Chl-a and is given by:

1 $$Ch-a={10}^{(0.2511-2.0853R+1.5035{R}^{2}-3.1747{R}^{3}+0.3383{R}^{4})}$$

where

2 $$R ={log}_{10}\frac{Rrs(490)}{Rrs(555)}$$

where:

*Ch-a* is the estimated quantified value of chlorophyll-a.

*R* is the quantifying coefficient derived from the spectral bands.

*Rrs* is Reflectance in the specified wavelength region.

For OC-2, the ratio of Rrs at Blue band at wavelength 490 nm and Rrs Green band at 555 nm are used to estimate the Chl-a as reported in the following dates.

#### Lake surface air temperature estimation from landsat 8 OLI

As another proxy of HABs^39^, this part of the methodology workflow therefore intends to estimate the LSAT using the Mono-window (Single-channel) algorithm and *in-situ* sensors.

Thermal conditions favor the growth, stability and spread of a wide variety of algal species^40,41^. Further, after their domination and colonization, these microscopic and photosynthetically active organisms increase the LSAT of the region.

L8 TIR supplies two thermal bands (10 and 11) for the retrieval of LSAT. Wang et al.^42^ proposed the Mono-Window algorithm for the retrieval of land surface temperature using the landsat 8 band 10. Moreover, this algorithm scores equally acceptable when applied in the retrieval of LSAT in inland water bodies, hence the adoption of the same in this study. The study uses Landsat 8 Collection 2 Level-2 surface temperature products for band 10, which are pre-processed by the U.S. Geological Survey (USGS) to include atmospheric correction, accounting for water vapor and other atmospheric effects critical for accurate LSAT estimation over water bodies^34^. For multispectral bands 2–5, used in parallel for chlorophyll-a (Chl-a) retrieval, Collection 2 Level-2 surface reflectance products were employed, which incorporate atmospheric correction optimized for aquatic environments.

The information acquired is processed in a multi-software platform, to calculate the lake surface air temperature. For this purpose, R programming environment was used.

For the successful retrieval of LSAT, the study adopts the following major steps alongside the supplied equations:

I. Conversion of L8 DN pixel values to at-sensor spectral radiance (Lλ)
II. Transformation of at-sensor spectral radiance to at-sensor brightness temperature (Tb10)
III. Estimation of lake surface emissivity
IV. Estimation of Lake Surface Air Temperature by adopting MW algorithm (Wang et al.^42^).

The Level-2 surface temperature products for band 10 provide atmospherically corrected data, reducing the need for additional correction in steps I and II above. However, to ensure consistency with the Mono-Window algorithm, the study verifies the spectral radiance and brightness temperature calculations using the following equations, which align with the Level-2 processing pipeline.

The first step is to convert the raw DN (Digital Number) values of band10 to obtain the TOA (top of atmosphere) spectral radiance ($L\lambda$) by multiplying the multiplicative radiometric rescaling factor (ML) of TIR bands with its corresponding TIR band and adding additive rescaling factor (A_L_) using Eq. (3) below:

3 $${\text{L}}\lambda = {\text{ML}}*{\text{QCal}} + {\text{A}}_{{\text{L}}}$$

Where:

$L\lambda$ is the spectral radiance in $\frac{watts}{({m}^{2}{srad}^{-1}{um}^{-1})}$

ML is the Band_10_ multiplicative rescaling factor obtained from metadata (e.g.,0.0003342);

QCal is the DN value for the quantized and calibrated standard product pixel of band 10.

A_L_ is the band-specific additive rescaling factor obtained from the metadata (0.1);

The obtained spectral radiance above is then used to retrieve brightness temperature (Tb_10_) in degrees Celsius, which is the Electromagnetic Radiance traveling upward from the top of the Earth’s atmosphere. Therefore, The Brightness Temperature is not a temperature on the ground rather is the temperature at the satellite^43^. The spectral radiance value obtained in Eq. (3) above, was then converted to brightness temperature BT by adopting Eq. (4) below:

4 $${\text{Tb}}_{{{1}0}} = \frac{K2}{{Ln\left[ {\left( {\frac{K1}{{L\lambda }}} \right) + 1} \right]}} - 273.15$$

where:

Tb_10_ is the brightness temperature in °C;

K1 and K2 are thermal constants, obtained from the metadata file of the L* OLI;

Lλ is top of atmospheric radiance.

#### Normalized difference vegetation index-NDVI

To later determine Lake surface Emissivity, NDVI; which is a mathematical algorithm that ranges between −1.0 to + 1.0 is essential to identify different land surface cover types of the study which is further necessary to calculate proportional vegetation (Pv) and lake surface emissivity (ε)^43^. NDVI is calculated on a per-pixel basis as the normalized difference between the red band (0.64—0.67 mm) and near infrared band (0.85–0.88 mm) of the images using the formula in Eq. (5) below.

5 $$\text{NDVI}=\frac{NIR-RED}{NIR+RED}$$

The NDVI for the study area derived from Eq. (5) above was then used to estimate the thermal emitting target under each lake surface cover type denoted as proportional vegetation (Pv) which is a direct function of NDVI.

The vegetation and bare soil proportions are acquired from the NDVI of pure pixels. Pv was calculated using the Eq. (6) below.

6 $${\text{Pv}=\left[\frac{NDVI - NDVImin }{NDVImax- NDVImin}\right]}^{2}$$

The proportion of vegetation obtained from Eq. 6 above is a key ingredient of calculating the Lake Surface Emissivity (ε), which is the radiative properties of lake features/targets which characterizes the ability of a body to emit thermal radiation energy across the lake surface into the air atmosphere. It is used to measure the capacity of lake water surface reflectance in the shortwave radiation band, which controls the energy partitioning between atmosphere and lake^44^. The knowledge of lake surface emissivity is further exploited to estimate lake surface air temperature.

7 $$\varepsilon = 0.00{4 }*{\text{ Pv}} + 0.{986}$$

Using the Lake Surface Emissivity (ε) derived above, we finally retrieve the required LSAT using brightness temperature (BT) obtained from band 10.

LSAT has a linear relationship with at sensor brightness temperature and Lake Surface Emissivity and can therefore be retrieved using the Eq. (8) below:

8 $$\text{LSAT}=\frac{Tb10}{\{1 + [(\lambda Tb10)]|\rho * Ln(\varepsilon )\}}$$

where,

LSAT is the LSAT in Celsius (˚C),

Tb10 is at-sensor BT (˚C),

λ is the average wavelength of band 10,

ε is the emissivity calculated from Eq. (7) above.

This methodological workflow algorithm finally derives Lake Surface Air Temperature for the region under study and the results were as presented in results section below.

#### Automated In-situ internet of things system

The automated *in-situ* system consists of a variety of hardware and software that work synergistically to collect and disseminate LSAT remotely.

The internet of things, conveniently referred to as IoT, is a network of interconnected computing devices, mechanical and digital machinery or people having unique identifiers (UIDs) and the ability to transfer data without requiring human-to-human or human-to-computer interaction^45^.

Narrowing down into the specifics of this research project, the word IoT has been used to refer to a man-made system that has been assigned a unique and private Internet Protocol (IP) address, loaded with sensors and is able to transfer data e.g.,

- Lake surface air temperature
- Lake surface salinity
- GPS location of the system at the time of the collection of such data
- The whole system in-house status including working conditions.
- Unexpected system failure
- System power level.

This system was deployed as an immobile in-situ setup for continuous near real-time LSAT monitoring at select locations in Lake Victoria prone to early HAB occurrence, ensuring consistent data collection from fixed points known for bloom initiation.

#### Devices and sensors used

The IoT system is built around a single board computer (SBC), specifically the Raspberry Pi Model 3B + , sourced from Nerokas Engineering Solutions, Kenya^46^. This SBC integrates a microprocessor with memory and circuits to control embedded system functionalities, such as the water quality monitoring system.

#### Reason for choice of raspberry Pi in this research

1. Higher processing power: Having the capacity to perform relatively similar as a full-blown computer, at 1.4 GHz quad-core ARMv8 64bit processor, it ranks the highest among microcomputers at the time of submission of this thesis by being able to simultaneously run multiple tasks at a go and further operate as a server gives it more potential score and rating above other microcontroller IoT single boards like Arduino, ESP32.
2. Little to no electronics prerequisites needed: Little prior embedded programming languages knowledge and its components is needed here. Unlike for Arduino and other ARM Cortex MCUs, one does not necessarily need to possess a very good and deep electronic background, and therefore no need to know about embedded programming languages e.g., Object oriented C + + .

#### Sensors used

The IoT system integrates sensors for LSAT and GPS data collection, with specifications detailed in Table 2 below.

**Table 2:** Specifications of IoT-integrated components for HAB monitoring.

Component	Function	Technical specifications
Raspberry Pi model 3B +	Single-board computer for data processing and system control & monitoring	1.4 GHz quad-core ARMv8 64-bit processor, operates as a server, runs Raspberry Pi OS
DHT11 air temperature sensor	Measures lake surface air temperature (LSAT)	Range: 0–50 °C, Accuracy: ± 2 °C, Input Voltage: 3.3–5.0 V, Requires 10KΩ pull-up resistor and filtering capacitor
Neo-6 M-ublox GPS sensor	Collects (x, y) coordinate location data	Receives GPS signals at 1575 MHz, provides precise location for LSAT data collection

## Lake surface air temperature (DHT11 sensor)

The DHT11, sourced from Nerokas Engineering Solutions, Kenya, is a widely accepted, relatively cost-effective and easy-to-use digital air temperature sensor.

### Features of DHT11 air temperature sensor

- Measures air temperature within the ranges 0–50 °C with an accepted accuracy of ± 2 °C.
- The sensor needs to be interfaced with a filtering capacitor and a pull-up resistor of 10KΩ connected in series with the VCC output pin of the sensor.
- Input voltage is ~ 3.3 V to 5.0 V.

### Mode of operation of DHT11 air temperature sensor

The DHT11 basically measures the Air Temperature under the concept that, with increase in the air temperatures, the resistance of the Negative Temperature Coefficient, NTC thermistor installed in it decreases, hence recording higher temperature values^47^

## Global positioning system (GPS) sensor

The *Neo-6 M-Ublox GPS* sensor module also sourced from Nerokas Engineering Solutions was settled on to solely play the role of collection of the (x, y) coordinate location information to pinpoint the system’s location during LSAT data collection and dissemination.

### Basic mode of operation

A basic GPS signal receiver capable of collecting the GPS pseudo frequency signals broadcast at 1575 MHz from GPS space segments and computationally determine the location of any system that it’s embedded to. This will tell us precisely where in Lake Victoria there exists abnormality in temperature rises, calling for an emergency mitigation action.

### Working and operation of the automated IoT system

An IoT ecosystem integrates an array of smart devices that use embedded systems technology, such as:

- In-built processors (e.g., Broadcom BCM2837 in the Raspberry Pi).
- An array of sensors (as previously discussed above) that collect and send the data from their point of attachments.
- Communication hardware; that disseminates the collected data.

**Fig. 3:**
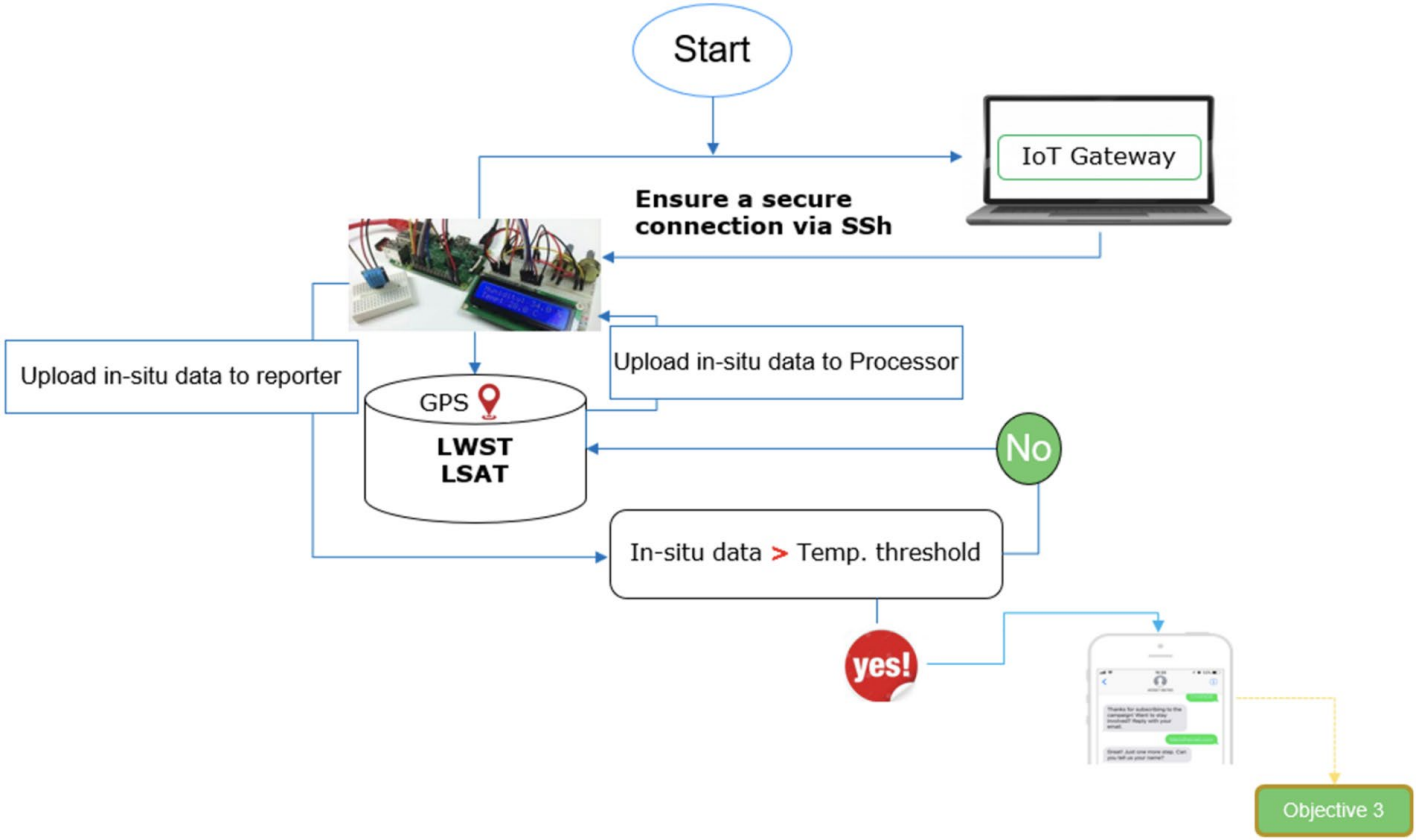
Overall methodology workflow for the IoT system.

Upon establishing a secure connection with an IoT gateway, sensors transmit data to a cloud server using POST/GET functionalities or process it locally. The system operates on Raspberry Pi OS, with Python scripts managing sensor data collection, processing, and transmission, leveraging libraries like Adafruit_DHT for DHT11 and pynmea2 for GPS data parsing. This fully automated setup, illustrated in (Fig. 3), requires minimal human intervention and was chosen over alternatives like Arduino for its superior processing capabilities and ease of software development.

### Accuracy assessment

To evaluate the reliability of the modelled Chl-a values, corresponding validation data were retrieved from ground truthing data collected by KMFRI and some from NASA Giovanni Chlorophyll-a portal, managed by Nasa Earth Data. To further assess how well the modelled LSAT match the reference data, validation data retrieved from NASA’s MODIS which collects global Sea Surface Temperature on a near-daily basis as one of its products. This would provide a quantitative evaluation of the adopted methodology for the study area for the years 2015 through 2021.

The statistical metric used for validation of both Chl-a and LSAT were the coefficient of determination (R^2^) as described in equations S1 and S2 (see Appendix A), along with root mean square error (RMSE), mean square error (MSE) and mean absolute error (MAE). These metrics provide standardized measures of predictive accuracy, with RMSE and MAE expressed in the same units as Chl-a, facilitating direct comparisons with other models, while MSE enhances sensitivity to outliers, critical for water quality monitoring. To ensure consistency with ground-truth and reference data, the Chl-a and LSAT data used for validation were not normalized, as raw values from Landsat 8, Sentinel-3 OLCI, and MODIS datasets were directly compared which lead to a simpler workflow due to reduced computational complexities and potential methodological bias. For Chl-a, R^2^ values ranged from 0.837 to 0.899 for Sentinel-3 OLCI and 0.667 to 0.821 for MODIS datasets. For LSAT, R^2^ values similarly indicated strong agreement with reference data, confirming the reliability of the methodology.

## Results

### Chl-a estimation

#### Chl-a concentration

The OC-2 algorithm was used to estimate HAB concentrations, with results presented both in the maps and tables below. For the study period (2015–2020), single Landsat 8 images were analyzed for specific dates when HAB events were reported by the Maritime Authority, as detailed in Table 1 of Section “Data”, Data. These dates were chosen to validate the spatiotemporal detection of HABs, with one image per year used to capture bloom events. The same images were used to estimate LSAT for the respective dates. All the resulting maps in this study were generated by authors using QGIS 3.40 platform, freely downloadable from https://qgis.org/.

**Fig. 4:**
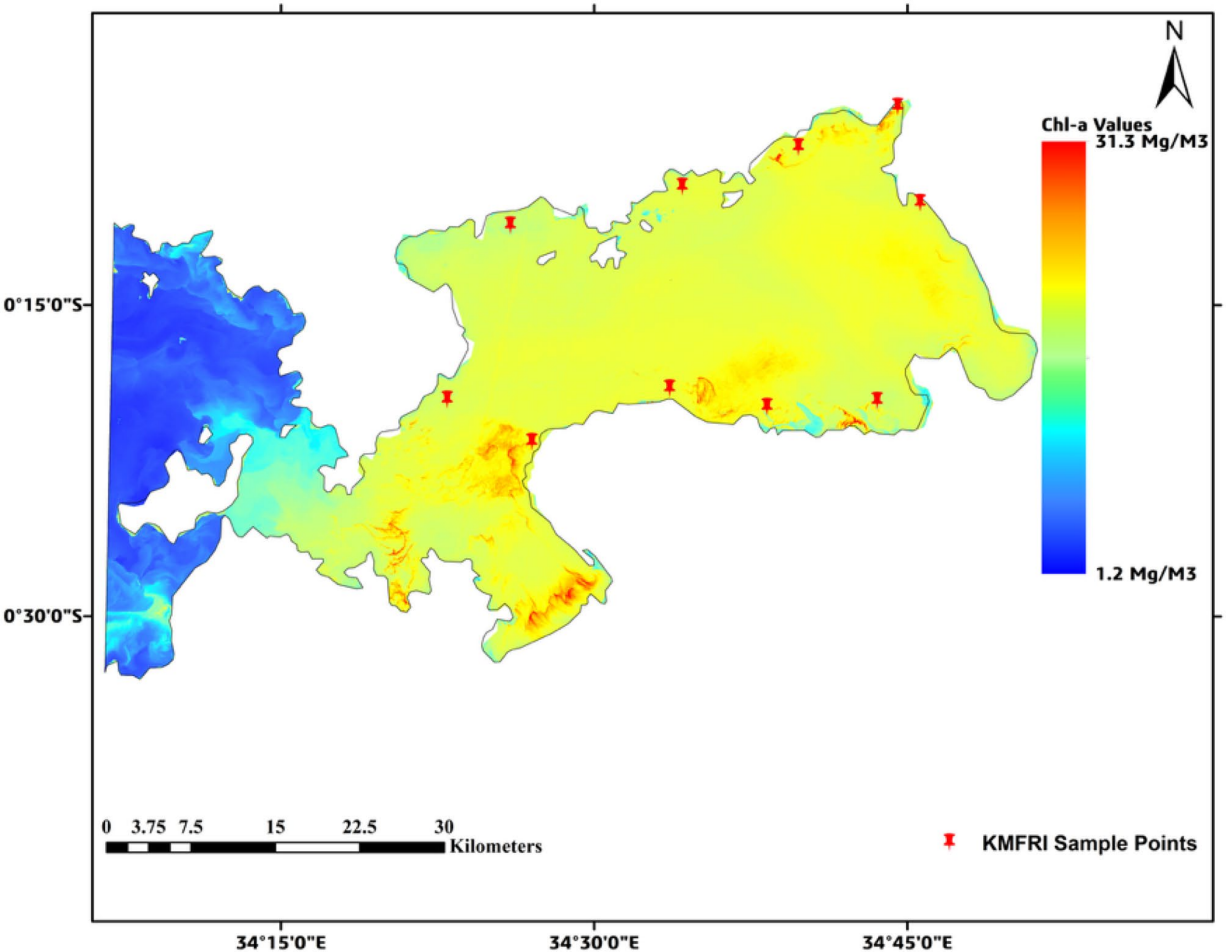
HAB concentration for 2015. Map by QGIS 3.40 (https://qgis.org).

**Fig. 5:**
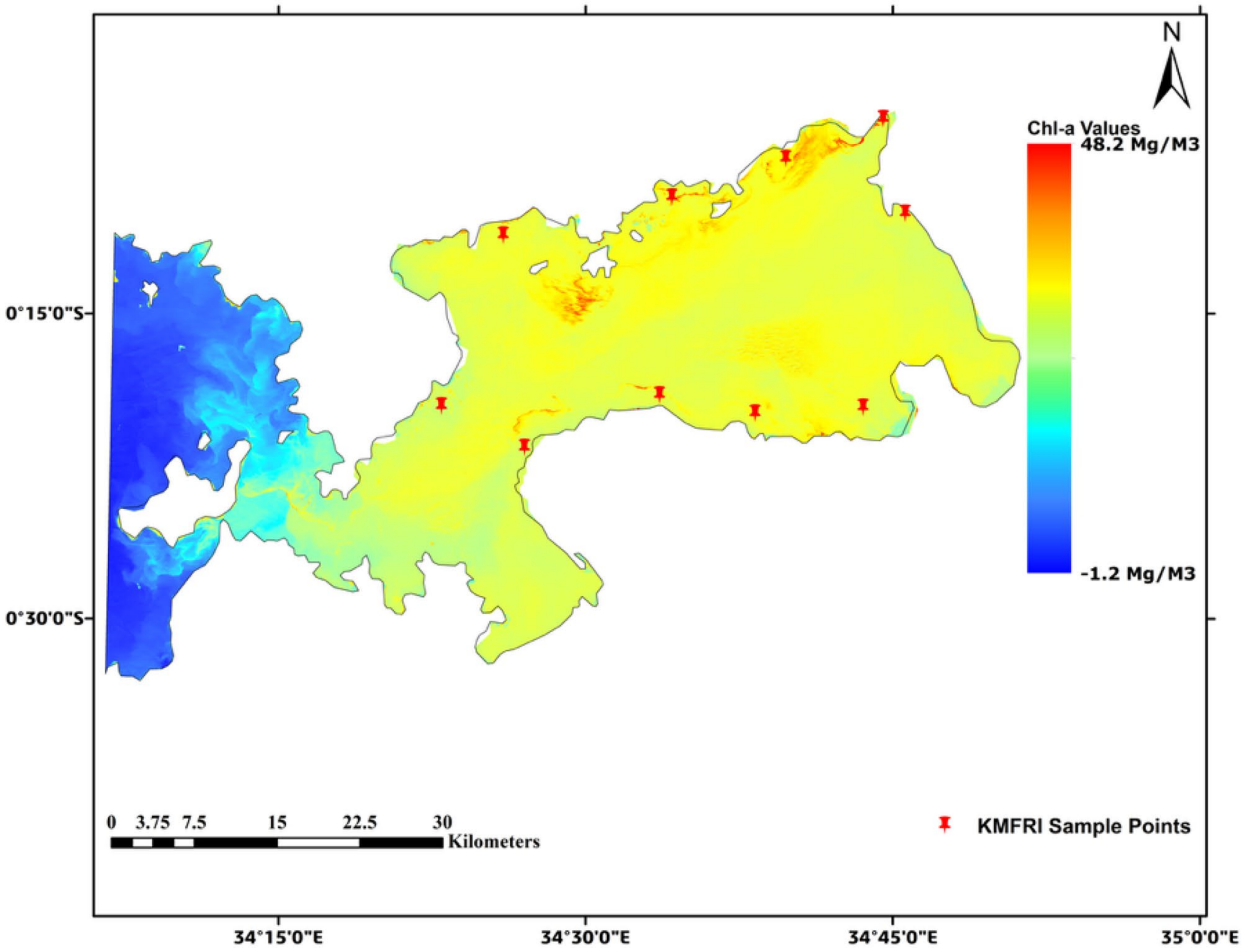
HAB concentration for 2016. Map by QGIS 3.40 (https://qgis.org).

In Figs. 4, 5 below, it is noted that on the event of HAB in 2015, Chl-a values rose significantly to up to 31.3 mg/m^3^ and 48.2 mg/m^3^ respectively in the affected regions as seen in red patched areas. This was unlike the case in the *blue* regions in the western parts of the study area as shown below. They (the western parts of the study area) reported very low Chl-a score around 1.2 mg/m^3^ especially for a non-turbid water body like Lake Victoria.

Other Chl-a levels were as shown in the Table 3 below, with their corresponding results map detailed in Figs. S1–S4 in the Appendix B section.

**Table 3:** Summarized Chl-a levels for years 2015 to 2020.

Year of HAB occurrence	Low Chl-a level ( mg/m^3^)	Highest Chl-a level (mg/m^3^)
2015	1.2	31.3
2016	−1.2	48.2
2017	16.4	39.6
2018	16.0	25.9
2019	16.1	49.3
2020	14.4	57.1

### LSAT estimation

#### LSAT For corresponding reported HABs events

As indicated in the methodology in Section “Materials and methods” and illustrated in (Fig. 3), the Mono-Window algorithm was used to estimate LSAT as another proxy for HABs, as reported. The same single Landsat 8 images obtained for the reported dates used for Chl-a estimation were analyzed to derive LSAT values.

**Fig. 6:**
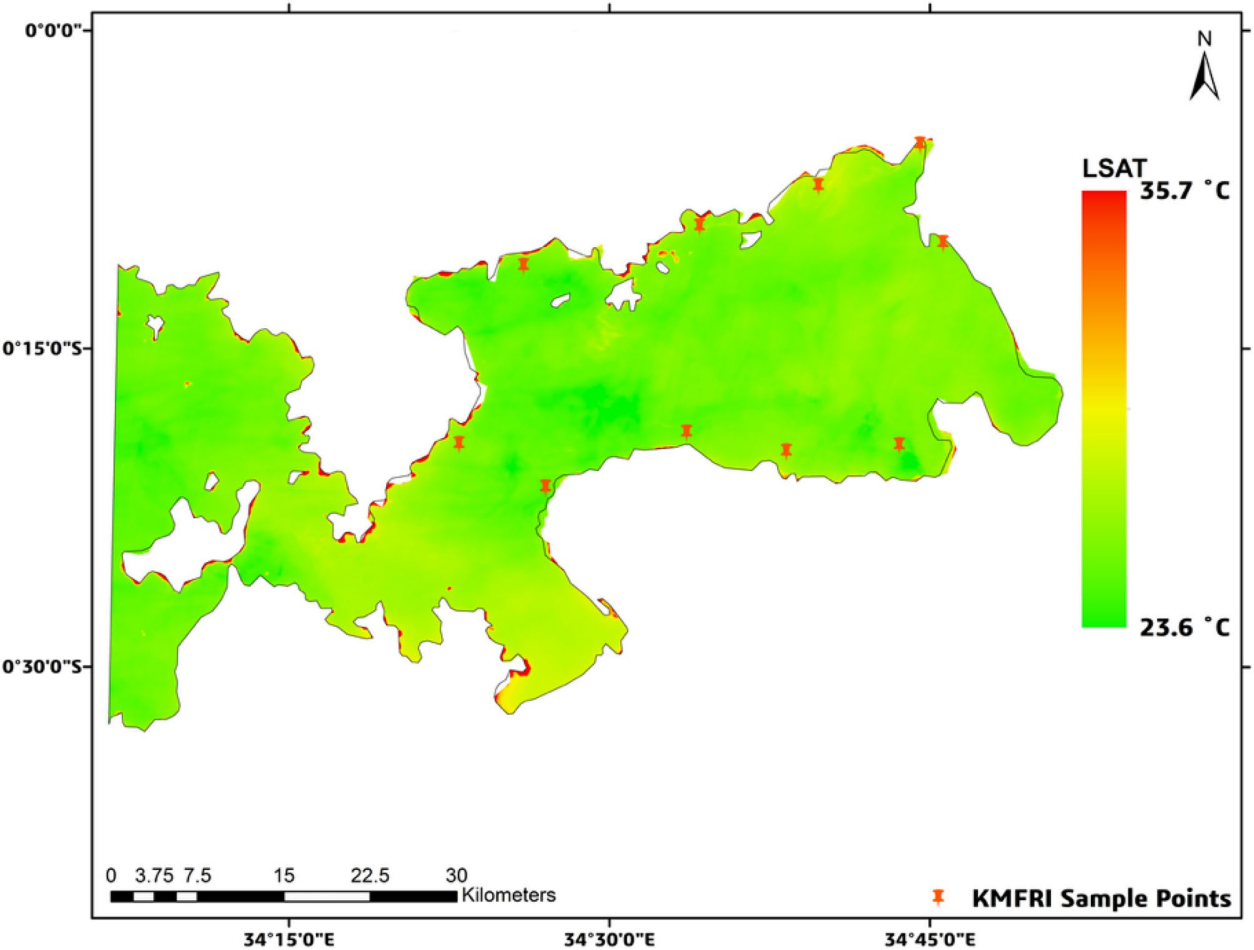
LSAT concentration for 2015. Map by QGIS 3.40 (https://qgis.org).

**Fig. 7:**
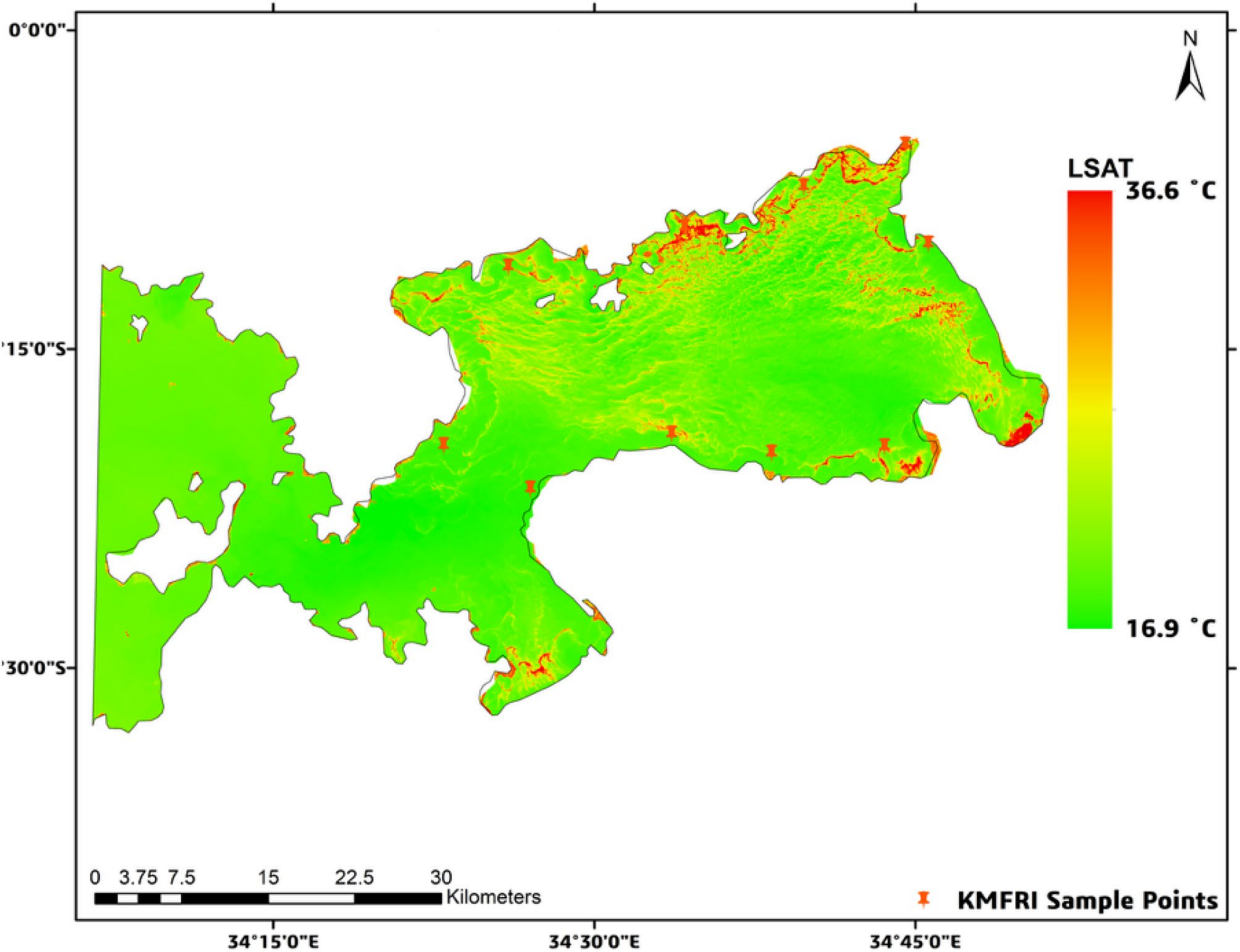
LSAT concentration for 2016. Map by QGIS 3.40 (https://qgis.org).

In Figs. 6, 7 below, it can be observed that in the event of HAB as reported, there existed a relatively high thermal emissivity from the Lake surface especially in the affected regions with LSAT values rising significantly to up to 35.7 °C in 2015 and 36.6 °C for 2016 especially in the affected regions as shown in the figures below. The unaffected regions however maintained low LSAT values of 23.6 °C and 16.9 °C respectively.

The same phenomen was manifested for the remaining years as summarised in Table 4 below. For ease of readability, other results are extensively illustrated in Figs. S5–S8 in the Appendix C section below.

**Table 4:** Summarized LSAT levels for years 2015 to 2020.

Year of HAB occurrence	Lowest level of LSAT level ( °C)	Highest level of LSAT level ( °C)
2015	23.6	35.7
2016	16.9	36.6
2017	26.2	35.1
2018	25.9	36.6
2019	28.7	36.8
2020	26.1	34.8

#### Accuracy assessment of the Chl-a estimates

To validate the estimated chlorophyll-a (Chl-a) results and analyze the relationship between Sentinel-3 OLCI Chl-a data and those retrieved from Landsat 8 OLI, scatter plots and linear regression were performed using 500 to 1000 samples of Chl-a values and their corresponding Sentinel-3 values. Results are summarized in (Table 5), with detailed scatter plots provided in Figs. S9–S12, of the supplementary Appendix D.

**Table 5:** Summarized accuracy assesment of landsat 8 (L8) Chl-a and LSAT estimates against reference datsets(Sentinel-3 OLCI and MODIS) using R^2^, RMSE, MSE and MAE.

Year of HAB occurrence	L8 Chl-a estimates Vs sentinel-3 OLCI reference data				L8 LSAT estimates Vs MODIS reference data			
	R^2^	RMSE (mg/m^3^)	MSE (mg/m^3^)^2^	MAE (mg/m^3^)	R^2^	RMSE (°C)	MSE (°C)^2^	MAE (°C)
2015	0.837	2.767	7.659	2.148	0.667	2.064	4.259	1.674
2016	0.843	4.410	19.448	2.637	0.834	1.562	2.439	1.249
2017	0.892	2.583	6.670	1.869	0.697	1.394	1.944	1.072
2018	0.839	3.459	11.967	2.622	0.782	1.270	1.614	0.959
2019	0.899	2.531	6.406	1.675	0.821	2.418	5.847	1.728
2020	0.882	2.526	6.379	1.816	0.754	1.248	1.556	0.951

**Fig. 8:**
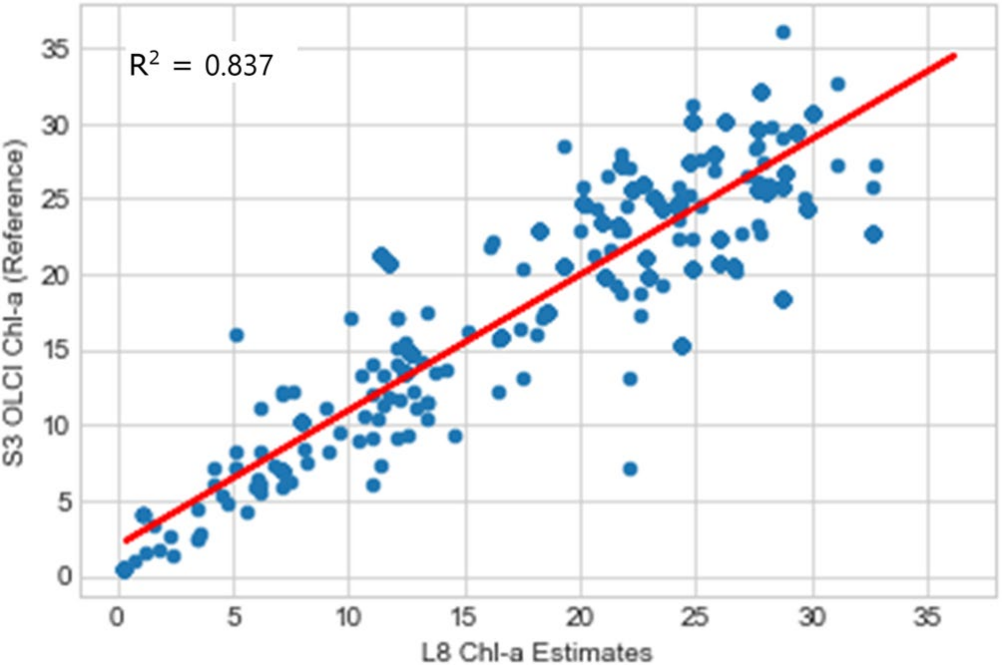
L8 OLI vs S3-OLCI Chl-a correlation for 2015.

**Fig. 9:**
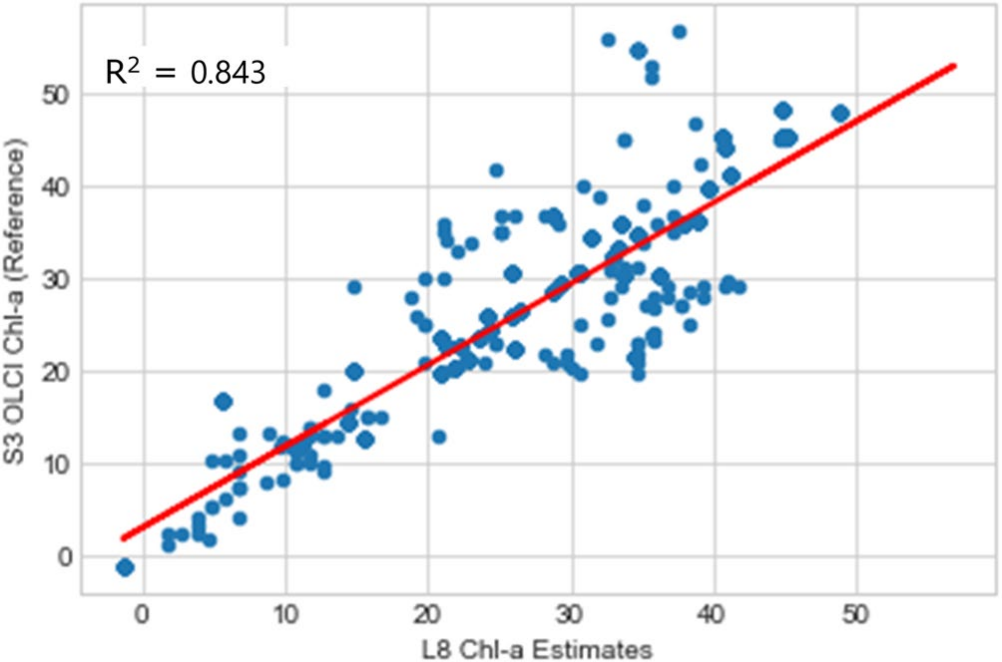
L8 OLI vs S3-OLCI Chl-a correlation for 2016.

Statistical analysis indicated (Figs. 8, 9) that the statistical coefficients of determination (R^2^) were seen to be 0.837 (2015) and 0.843 for 2016. Other corresponding accuracy assesmnet from coefficients of determination (R^2^) for subsequent years were as illustrated in Table 5 below.

#### Accuracy assessment of the LSAT estimates

To validate the estimated Lake Surface Air Temperature (LSAT) results and analyze the relationship between MODIS LSAT data and those retrieved from Landsat 8 TIR, scatter plots and linear regression were conducted using 500 to 1000 randomly generated samples of LSAT values and their corresponding MODIS values. The statistical metrics used were coefficient of determination (R^2^), RMSE, MSE and MAE. RMSE and MAE allow direct comparisons in LSAT units, while MSE highlights outliers relevant to water quality. Results are consolidated in (Table 5), with more graphical representations in Figs. S13–S16 in the appendix D section.

**Fig. 10:**
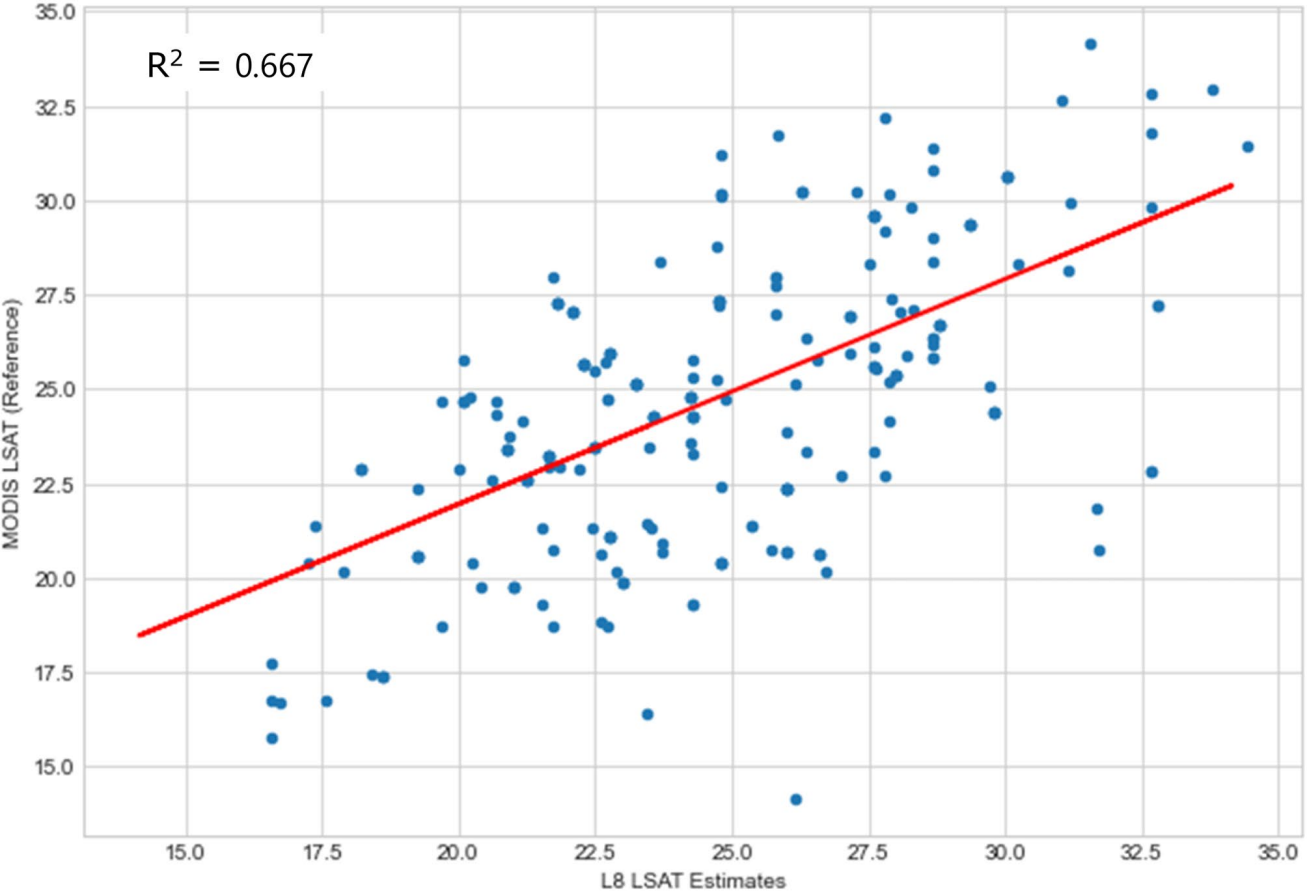
L8 TIRs vs MODIS LSAT correlation for 2015.

**Fig. 11:**
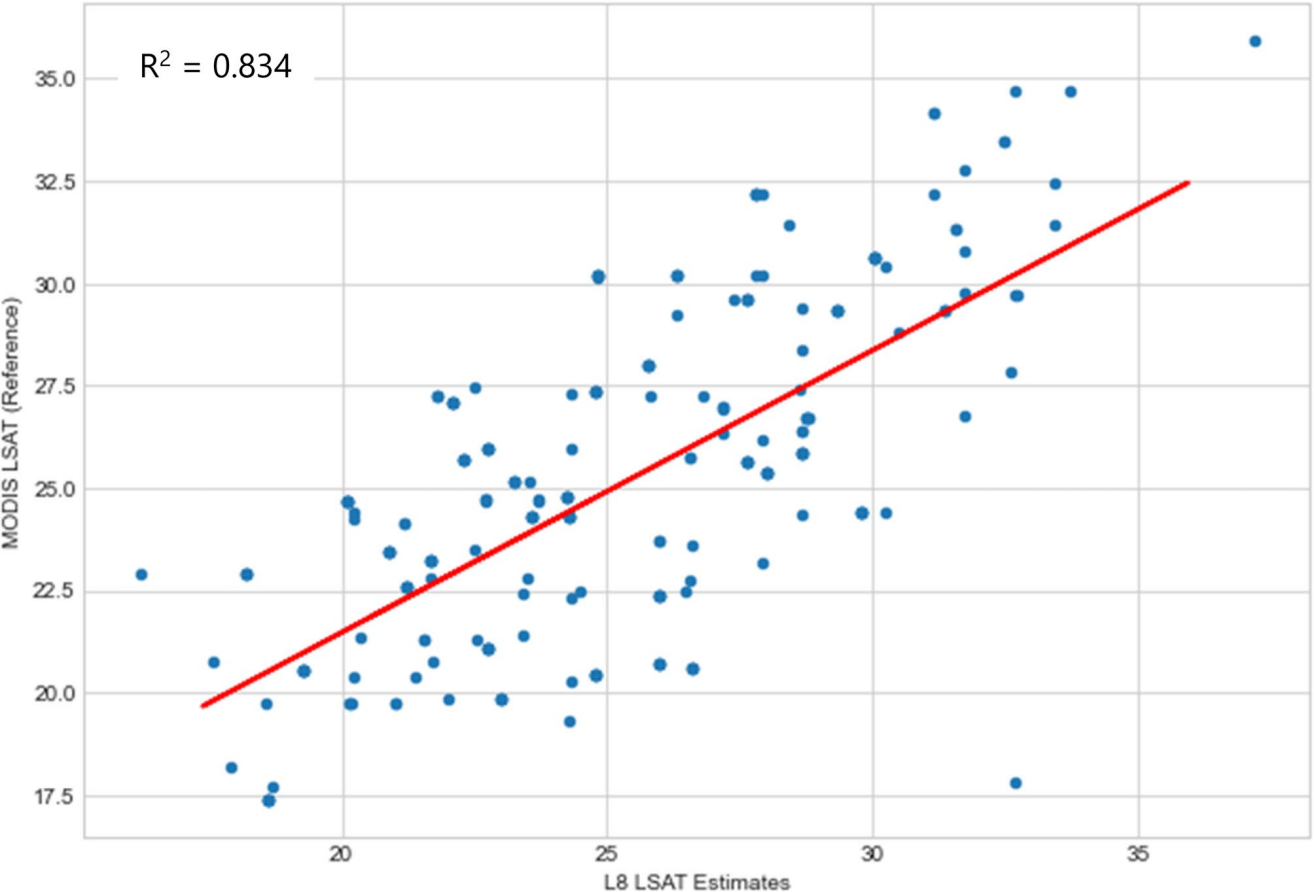
L8 TIRs vs MODIS LSAT correlation for 2016.

Statistical analysis showed (Figs. 10, 11) that the coefficients of determination (R^2^) were seen to be 0.667 (2015) and 0.834 for 2016. Other corresponding coefficients of determination (R^2^) for subsequent years were as illustrated in Table 5 below.

These correlations of determinations essentially implies that there did exist significant strong positive correlation between Lake Surface Air Temperatures estimated by Moving Window algorithm and NASA’s MODIS data products provided by the Earth Data.

In conclusion, regions with insufficient estimate parameters for Lake Surface Air Temperature, Moving Window algorithm can be reliably and accurately be applied to estimate using Landsat 8 TIRs.

### Automated in-situ IoT system

To come up with a near real-time ground-based monitoring system, an Automated In-situ IoT system was developed, tested, and found to be fully operational.

This was primarily to collect geotagged lake surface air temperature and lake surface pH, in this case the salinity being the case.

The automated IoT system which targets local fisheries authorities in the Lake Victoria region, responsible for overseeing water quality and fishing activities, automatically collects lake surface air temperature (LSAT) and sends geotagged SMS notifications to these authorities when LSAT exceeds pre-set thresholds ranging from 26 to 35.1 °C depending on the area of interest, indicating potential harmful algal blooms (HABs) conditions as identified in the study. The Africa’s Talking text message API facilitates near real-time delivery of these alerts, sent daily when thresholds are exceeded. The authorities subsequently advise local fishermen and communities on precautionary measures, such as restricting fishing or water use, based on the alerts.

**Fig. 12:**
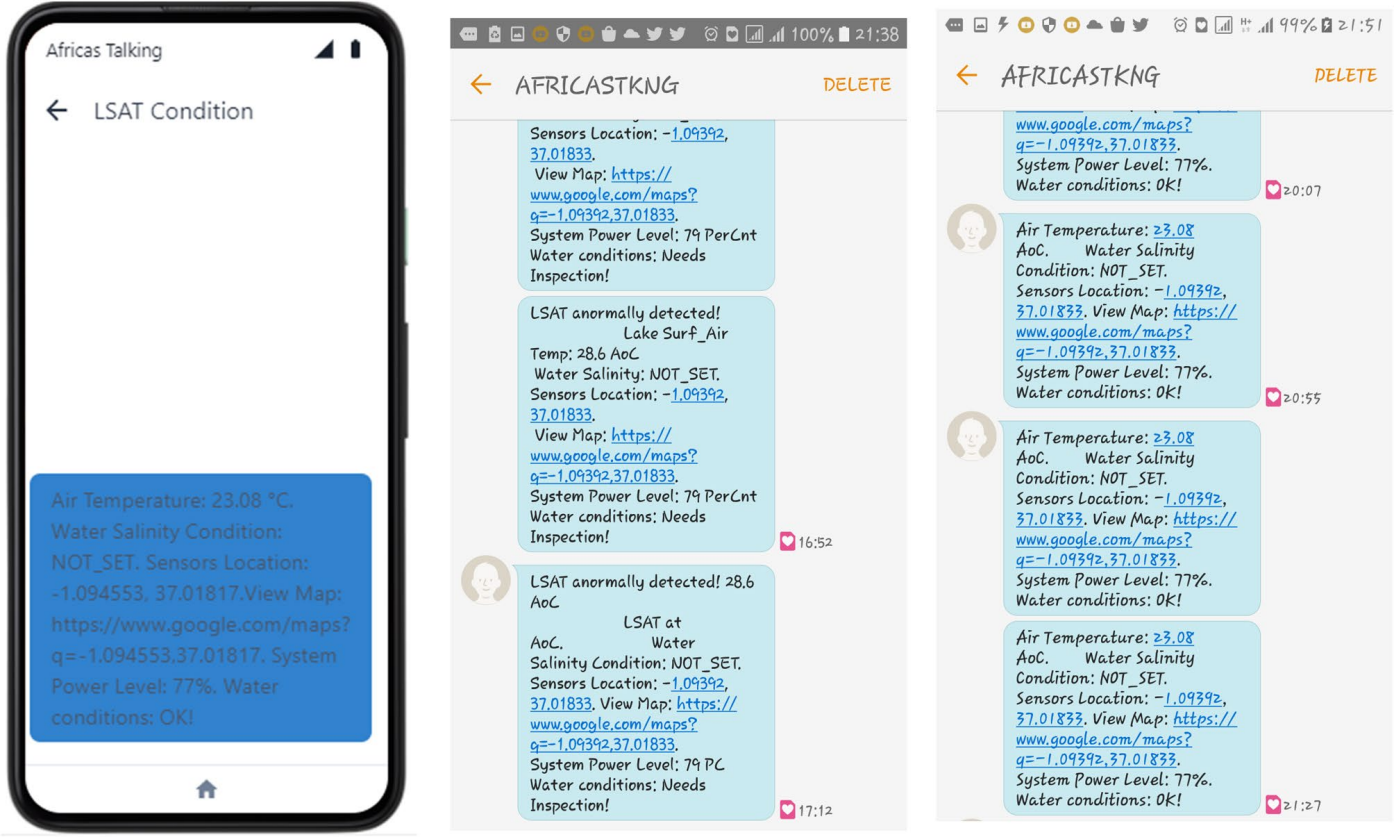
(**a**–**c**) The text message UI when received by client/user showing the IoT system sending SMS of the Geotagged LAST Conditions with the first image reporting a LSAT of 23.08 °C. Further the system provides a browser-viewable map link of the system’s location. Other water quality data were not set due to the unavailability of the sensor.

Figure 12a–c indicates how the message upon arrival appears in the GUI. The text comes along with a Google Maps link indicating the System location from which such data is coming from.

When the user clicks the link, a Google Map UI is realized, showing the Google Map being rendered on the users’ screen to show the real-time map locating the system.

## Discussion and recommendations

The findings from the methodological approach adopted, data, and results obtained in this extensive research, demonstrates that remote sensing which offers continuous, precise, and long-term datasets for surface water research greatly play a significant role in detecting and mapping of Harmful Algal Blooms in freshwater inland water bodies like Lake Victoria^48^. To further facilitate real-time monitoring, an IoT powered *insitu* system that was incorporated to complement the remote-sensed data by providing ground truthed data enhanced the monitoring and alerting capabilities.

From the reported bloom dates followed by the satellite imagery data analyzed from 2015 through 2021, the Chlorophyll-a (Chl-a) oncentrations observed in aquatic systems positively correlated to Harmful Algal Blooms (HABs) which are a result of massive proliferation of blue-green algae (Cyanobacteria)^49^,. Chl-a is a photosynthetic pigment which is present in all algal species and its presence is a direct indicator of HABs^50^, explaining the positive correlation.

Spatiotemporal analysis of the satellite imageries captured on the dates of the HAB occurrence showed that Chl-a concentrations were high and exceeding the minimum optimal value of non-turbid waters like Lake Victoria. The optimal Chl-a levels for non-turbid inland water bodies has been determined to range from 1.39 to 20.05 mg/m^3^ depending on sampling location, spatial heterogeneity water depth, human impact and natural variability like season and weather patterns^51^. The rise in the Chl-a levels took effect owing to the circumstantial elevated proliferation, predominance and therefore prevalence of the *Microcystis aeruginosa* species that produce the toxins^7^.

While nutrients like nitrogen (N) and phosphorus (P) are equally critical drivers of HABs^52^, this study focued on two directly detectable proxies through spatial remote sensing and IoT systems i.e. Chl-a and LSAT. Further, nutrient data requires intensive field sampling, which contrasts with the studies aim of developing a cost-effective but highly scalable monitoring framework.

Out of all the incidences, it was noted that 2018 exhibited the lowest magnitude of impact as manifested by the least Chl-a concentrations of 25.9 Mg/M^3^. 2020 bloom occurrence saw the highest concentrations of 57.1 Mg/M^3^. Generally, the highest concentrations in most cases were located either at the North, South or Central part of the Lake Victoria basin, without necessarily manifesting any predictable trend. The used Ocean Color-2 algorithm presented in this literature was able to effectively model and represent this phenomenon.

Some scholars like^53^ have however previously claimed that in some cases anthropogenic nutrients may not directly stimulate HABs, but may become linked to their growth and abundance. Our study confirmed that eutrophication, that is ascribed by a high discharge of pollutants coming from the busy industrial sections of the riparian reserves directly played the greatest responsibility for the proliferation of harmful algae. This correlation was equally observed by recent researchers like^54^.

Regarding the lake surface air temperature (LSAT), the values were seen to corroborate with the Chl-a values since high temperatures spots were as well associated with relatively high Chl-a concentrations. Lake surface temperature was noted to be an important factor contributing to lake HABs hence elevated Chl-a concentrations, and was equally seen to be positively correlated with Chl-a in almost all occurrences^55^. This agrees with the hypothesis that algae occurrences are closely associated with high temperatures and upon their occurrence, they further elevate the LSAT.

The estimated Chl-a and LSAT correlated strongly with the well accepted Sentinel 3 OLCI Chl-a (R^2^ scores ranging from 0.83 to 0.899) and MODIS LSAT products. Again, the estimated LSAT were correlated with the MODIS LSAT products (R^2^ scores ranging from 0.67 to 0.82). These strong positive correlations of determinations indicate robust model performance.

The IoT system, comprising a Raspberry Pi 3B + with DHT11 and Neo-6 M GPS sensors, provided real-time LSAT and location data (WGS84 latitude, longitude, GMT), displayed on remote handheld devices (Fig. 12a–c). Deployed at fixed locations prone to early HABs, this immobile system ensured consistent, high-frequency data collection, complementing satellite observations. Its automation, driven by Python-based software on Raspberry Pi OS, minimized human intervention, enhancing scalability and cost-effectiveness compared to traditional field sampling. This integration of IoT and remote sensing offers a novel framework for HAB monitoring, enabling rapid detection and response to blooms, though challenges remain in expanding sensor coverage and integrating additional parameters like salinity or nutrients.

### Recommendations

After the implementation of a variety of proposals, the following are areas of future research that need to be prioritized and scaled for future betterment of HAB monitoring, reporting and potential prevention:

- Increase the temporal resolutions of the monitoring and reporting, there need to do a Spatiotemporal image fusion of Landsat 8 and MODIS aqua to provide a near daily 30 M spatial resolution of Chl-a and LSAT estimates instead of waiting for the 16-day 30 M Landsat as this current study implements.
- Beyond the implemented IoT solution in the research, a more advanced and elaborate monitoring & alerting system should be prioritized to detect, predict, and alert the potential occurrence of HABs in real-time, thus enabling better prevention and management effort by the concerned authority.
- With climate change being an issue of concern, there is a need to enhance the suggested forecasting models, while considering the various climatic factors e.g.generally warmer water temperatures, changes in salinity dues to increases in atmospheric carbon dioxide concentrations and changes in rainfall patterns that may catalyze HAB formation, dominance, spread and their colonization.
- The Maritime and Environmental management authorities within the Lake Victoria riparian region should take more radical measures to ensure that the wastewater industrial discharges from the neighborhood are well treated before they are executed into the lake to minimize the eutrophication of the Lake Victoria basin.
- Further studies should incorporate additional *in-situ* water quality sensor such as water pH (Salinity), Secchi depth – water clarity, and turbidity as complementary indicators of HABs. Additionally integrating nutrient sensors e.g. phosphorus and nitrogen into the IoT network alongside the remote sensing data would enable a more comprehensive understanding of HAB triggers and dynamics, although this was beyond the scope of the current study.

## Conclusions

The study presented a methodological approach to detect and monitor the concentration of chlorophyll-a(Chl-a) from Landsat 8 Operational Land Imager data as HAB proxies in Lake Victoria from 2015 to 2020. This was implemented through the Ocean Colour 2 algorithm. Therefore, space-based observations play a significant role in the monitoring of Harmful Algal Blooms.

Further, Landsat 8 Thermal InfraRed (TIR) sensor aboard the same space vehicle was able to provide for corresponding thermal images to estimate the rising levels of Lake Surface Air Temperatures in the advent of HAB occurrence.

it was noted that in the occurrence of HAB, Chl-a values rose significantly to 31 to 57.1 mg/m^3^. Similarly, the moving window algorithm for LSAT estimates indicated that on a bloom event, the LSAT rose in the affected areas up to values such as 35.1 to 36.6 °C. Conversely, western parts of the study area that were never affected by the phenomena marked the lowest Chl-a value (−1.2 Mg/M^3^) and corresponding temperatures value (less than 27 °C).

With the unfortunate lack of synchronized measured ground truthing data from the authorities like KMFRI, the estimated Chl-a and LSAT products have been validated using in Chl-a products from Sentinel 3 -OLCI and LSAT with the MODIS (MOD11A1) data; Validation results from the scatter plots confirmed that the acceptably high correlation coefficient values of 0.743 to 0.876 for LSAT and 0.845 to 0.902 for Chl-a, this confidently demonstrates the applicability of the proposed and adopted algorithm in Chl-a estimation and LSAT retrieval in the study area.

Finally, to ensure near-real time monitoring at field level, an automated in-situ system was developed to collect and monitor the lake surface air temperature (LSAT). The system was tested and found operational and was able to report remotely in case of any abnormal rise in the Lake Surface Air Temperature, calling for further response. This would help the maritime authorities to take effective response to the alert sent and take corrective measures. The system was tested in the local University Pond as indicated in Fig. 12a–c and was able to report Surface Air Temperature values of ranges 23 to 28.6 °C.

## Supplementary Information

Supplementary Information.

## Data Availability

This study embodies data from two main sources; 1. The Kenya Marine, Fisheries & Aquatic Environment Monitoring Institute. 2. Freely available Large sized raw raster Datasets downloaded from USGS & Copernicus website. This first dataset includes spatial locations where relevant parameters such as “Lake Surface Air Temperature, Chl-a level, algal bloom presence, Dissolved Organic Content” were observed, reported and collected in alignment with the objectives of this study. Access to the relevant aggregated dataset that facilitated my findings is hereby granted, and have been deposited in this GitHub link: https://github.com/OkomoJacob/FinalYearProject/tree/main/HAB_MCC_DOC_Dates_Data. In relevance the weight of the raw spatial raster dataset, it was reasonably impossible for the author to publish the raw dataset. However the processed Spatial raster data is provided within the manuscript in the Results section.
