# Supplementary material for: Spatiotemporal modelling and monitoring of harmful algal blooms using IoT in Lake Victoria Basin Kenya
Source: Sci Rep. 2025 Oct 30;15:38053. doi: 10.1038/s41598-025-21979-3 (PMC12575628; doi:10.1038/s41598-025-21979-3)
Supplement: Supplementary file 1 — Supplementary Information. [file 41598_2025_21979_MOESM1_ESM.pdf]

Appendix A: Supplementary Equations; Statistical metrical coefficient of determination (R^2).

$$R^2 = \frac{\Sigma(Modeled_{Chl-a,i} - Mean_{Chl-a,i})^2}{\Sigma(Actual_{ActChl-a,i} - Mean_{ActChl-a,i})^2} \quad \ldots\ldots\ldots\ldots\ldots\ldots\ldots\ldots\ldots\ldots \textit{Eq. S1}$$

Where

$Modeled_{Chl-a,i}$ *is the estimated Chl-a at point i,*

$Mean_{Chl-a,i}$ *is the mean of the modeled Chl-a from Landsat 8 at point i,* $Actual_{ActChl-a,i}$*represents the reference Chl-a from Sentinel-3 OLCI at points i,* $Mean_{ActChl-a,i}$*while is the average of the sampled actual Chl-a from Sentinel-3 OLCI sensor.*

While the statistical metrics used for validation of LSAT was the coefficient of determination (R^2) as given by the equations below

$$R^2 = \frac{\Sigma\left(Modeled_{LSAT,i} - Mean_{LSAT,i}\right)^2}{\Sigma\left(Actual_{LSAT,i} - Mean_{LSAT,i}\right)^2} \quad \ldots\ldots\ldots\ldots\ldots\ldots \textit{Eq. S2}$$

Where:

$Modeled_{LSAT,i}$ is the estimated LSAT at point *i,*

$Mean_{LSAT,i}$ is the mean of the modeled LSAT from Landsat 8 at point *i*,

$Actual_{LSAT,i}$ represents the reference LSAT at points *I*,

$Mean_{ActLSAT,i}$ while is the average of the sampled actual LSAT from MODIS.

Appendix B: Other Results for Chl-a concentration from Year 2017 to 2020

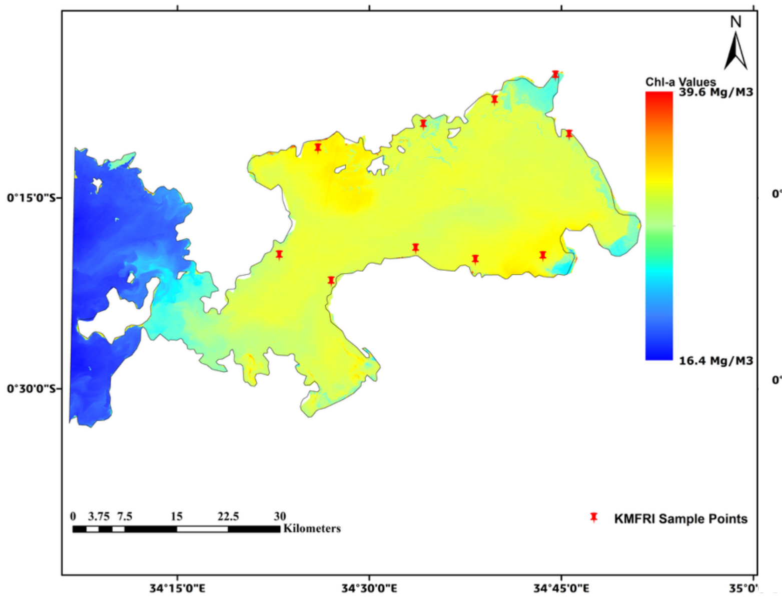

Fig. S1 *HAB concentration for 2017. Map by QGIS 3.40 (https://qgis.org)*

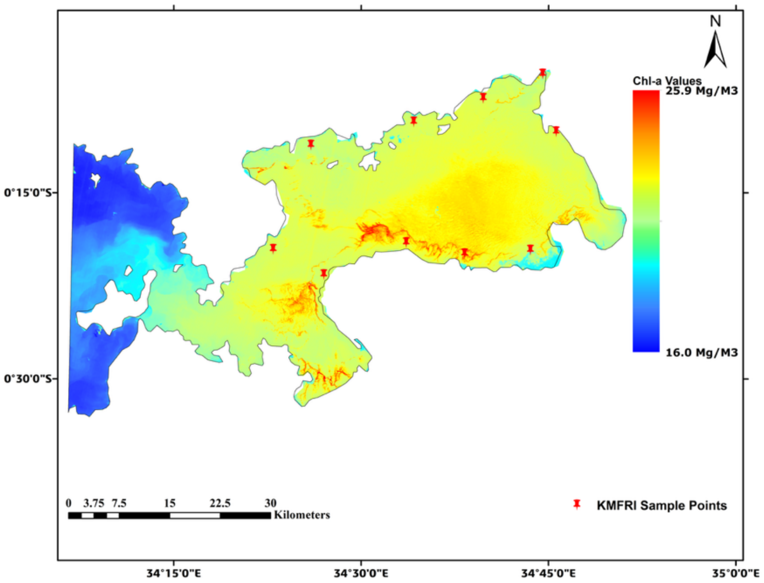

Fig. S2 *HAB concentration for 2018. Map by QGIS 3.40 (https://qgis.org)*

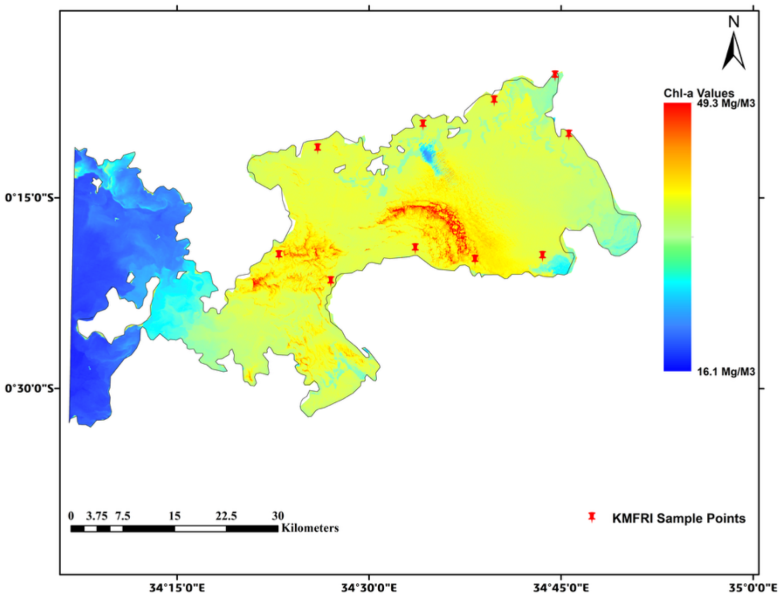

Fig. S3 *HAB concentration for 2019. Map by QGIS 3.40 (https://qgis.org)*

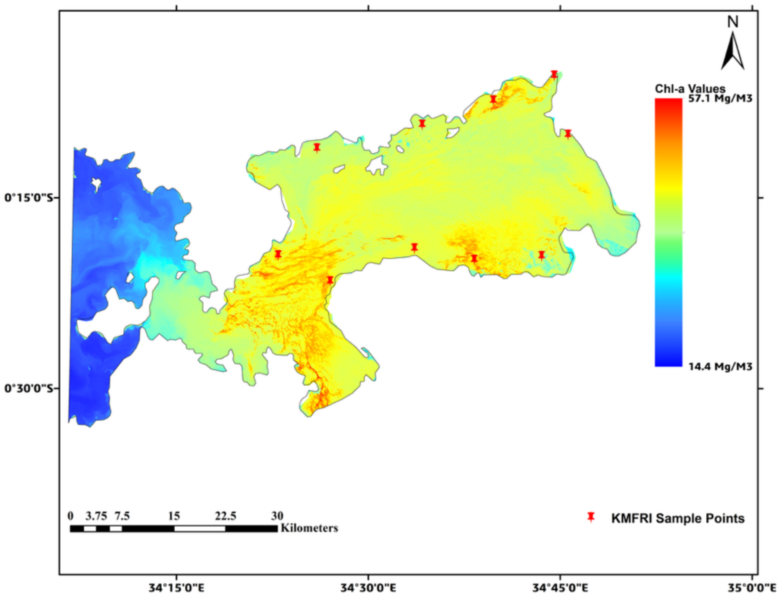

Fig. S4 *HAB concentration for 2020. Map by QGIS 3.40 (https://qgis.org)*

Appendix C: Other Graphical Results for LSAT Estimation from year 2017 to 2020

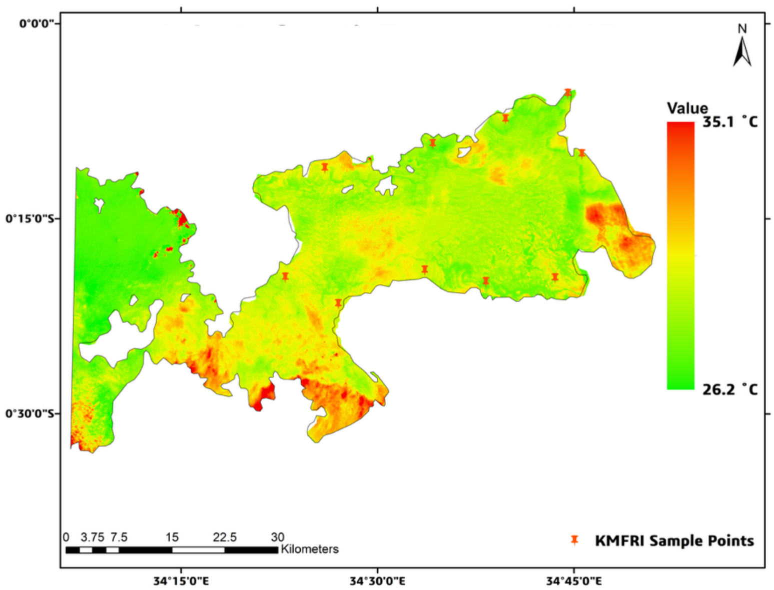

Fig. S5 *LSAT concentration for 2017. Map by QGIS 3.40 (https://qgis.org)*

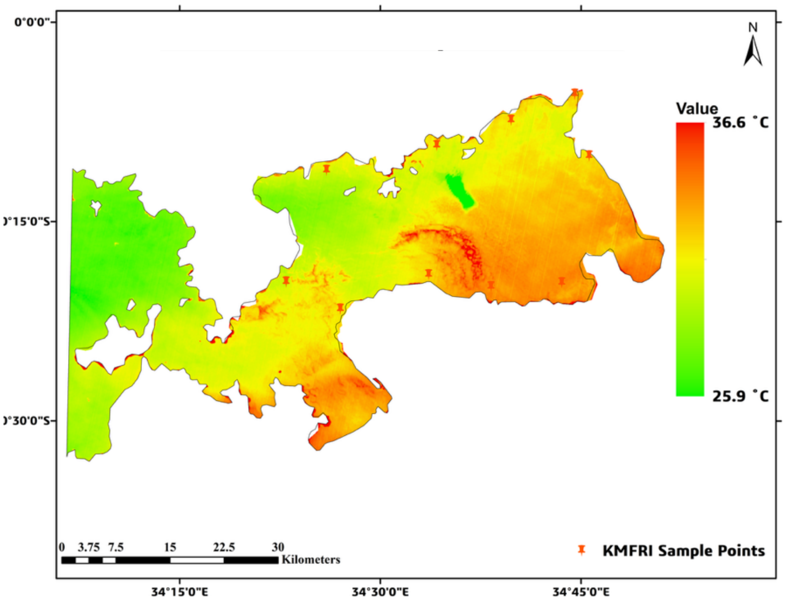

Fig. S6 *LSAT concentration for 2018. Map by QGIS 3.40 (https://qgis.org)*

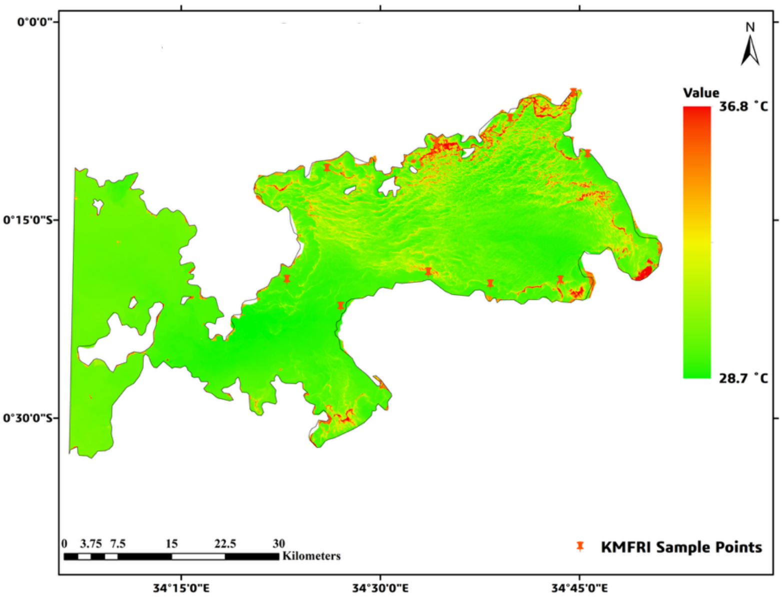

Fig. S7 LSAT concentration for 2019. Map by QGIS 3.40 (https://qgis.org)

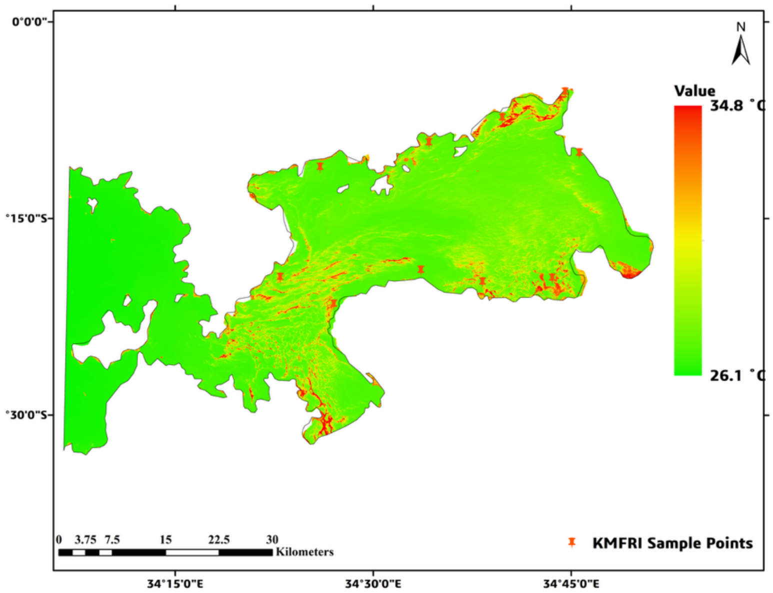

Fig. S8 LSAT concentration for 2020. Map by QGIS 3.40 (https://qgis.org)

Appendix D: Other correlation scatter plots for Chl-a & LSAT accuracy assesments from year 2017 to 2020

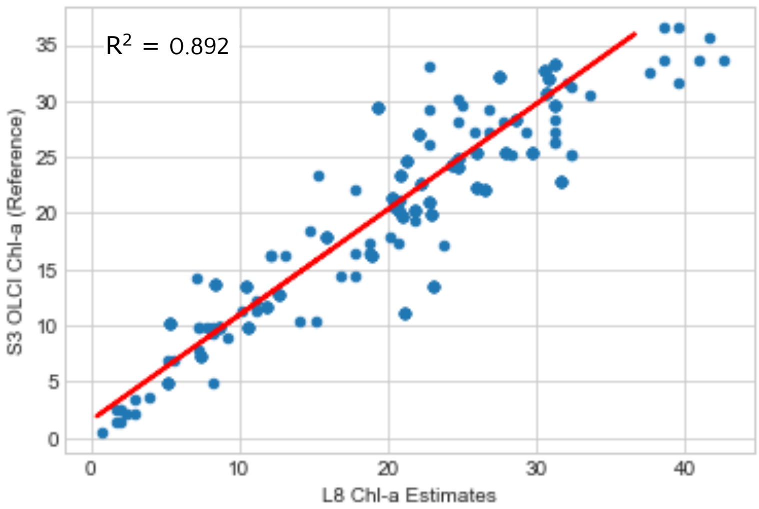

***Fig. S9** L8 OLI vs S3-OLCI Chl-a correlation for 2017*

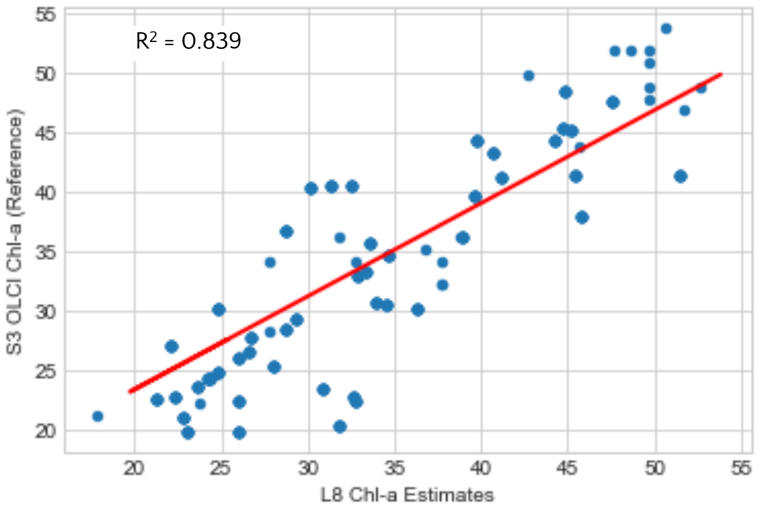

***Fig. S10** L8 OLI vs S3-OLCI Chl-a correlation for 2018*

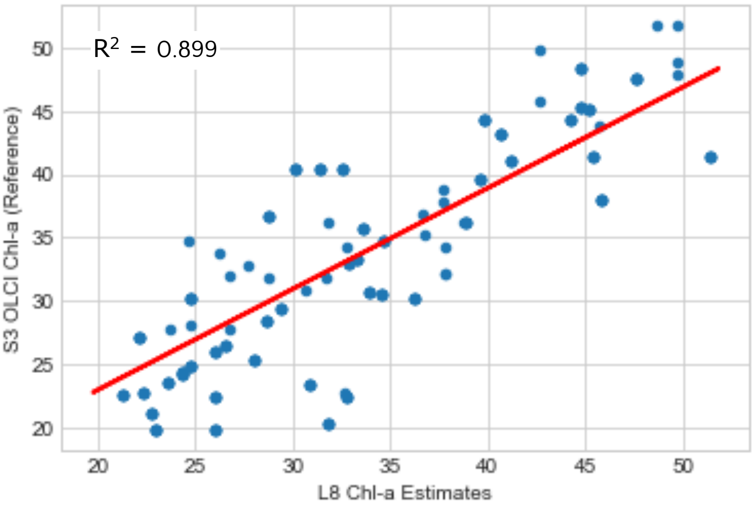

Fig. S11 *L8 OLI vs S3-OLCI Chl-a correlation for 2019*

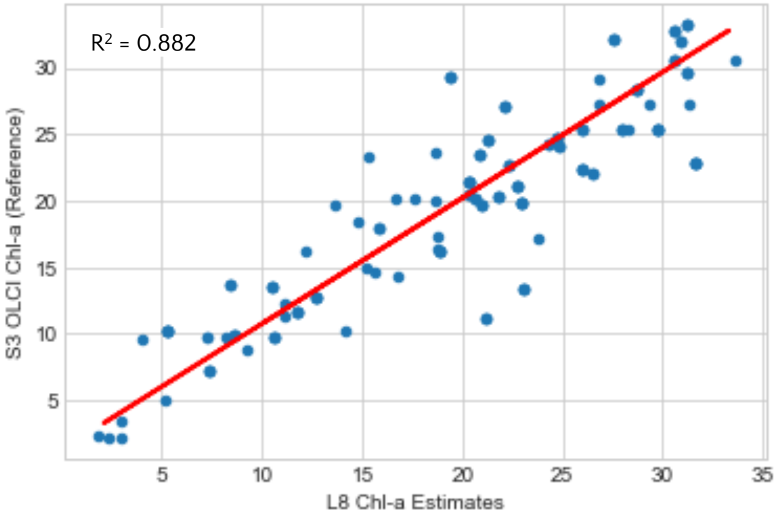

Fig. S12 *L8 OLI vs S3-OLCI Chl-a correlation for 2020*

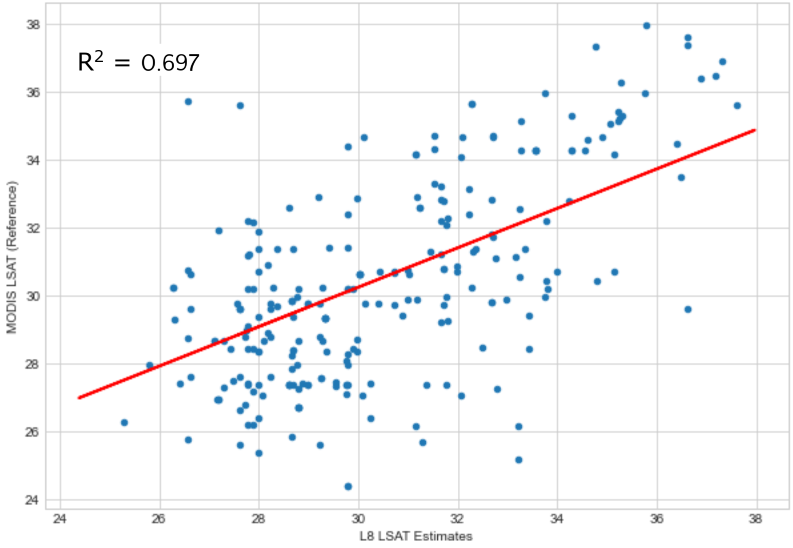

Fig. S13 *L8 TIRs vs MODIS LSAT correlation for 2017*

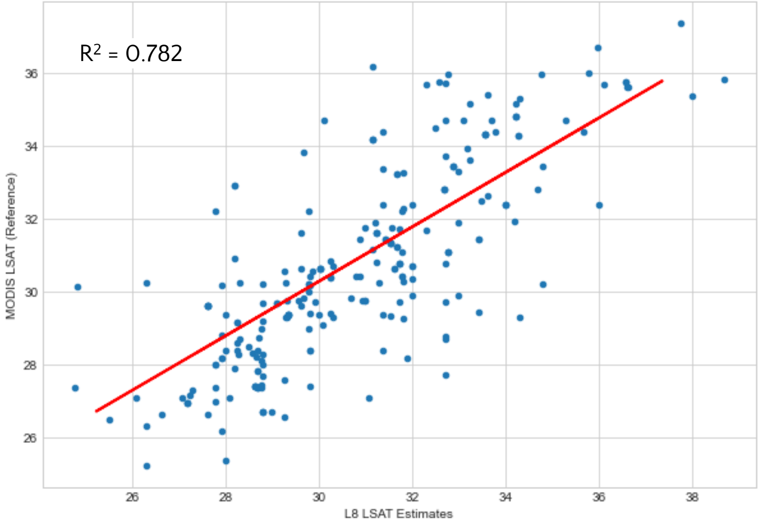

Fig. S14 *L8 TIRs vs MODIS LSAT correlation for 2018*

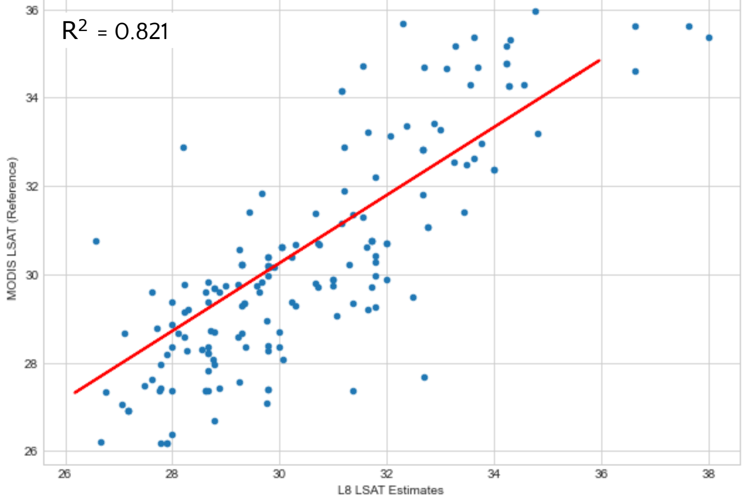

Fig. S15 *L8 TIRs vs MODIS LSAT correlation for 2019*

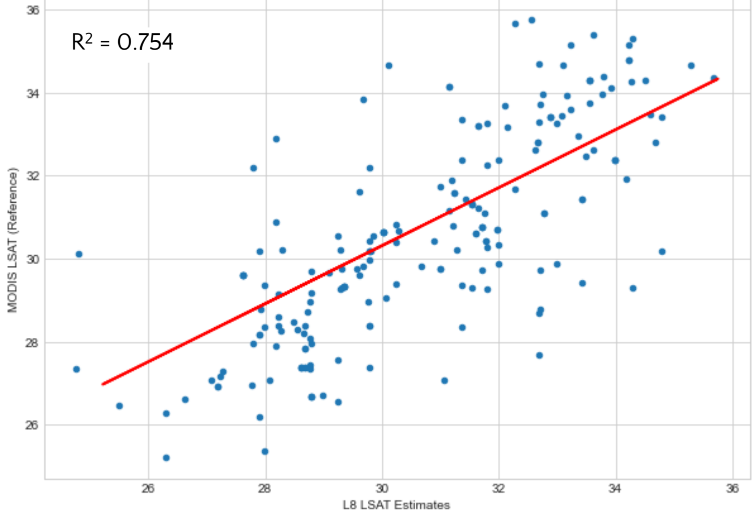

Fig. S16 *L8 TIRs vs MODIS LSAT correlation for 2020*
